# Synthesis Methods of Carbon Nanotubes and Related Materials

**DOI:** 10.3390/ma3053092

**Published:** 2010-05-07

**Authors:** Andrea Szabó, Caterina Perri, Anita Csató, Girolamo Giordano, Danilo Vuono, János B. Nagy

**Affiliations:** 1 Department of Chemical Engineering and Materials, University of Calabria, via P. Bucci, 87036 Arcavacata di Rende (CS), Italy; E-Mails: andrea.szabo@azet.sk (A.S.); cperri@unical.it (C.P.); csatoanita@gmail.com (A.C.); ggiorda@unical.it (G.G.); 2 NANOPART S.A., 60 Kapeldreef, 3001 Leuven, Belgium; E-Mail: danivuono@gmail.com (D.V.)

**Keywords:** carbon nanotubes, CVD, synthesis methods, laser ablation, arc discharge

## Abstract

The challenge on carbon nanotubes is still the subject of many research groups. While in the first years the focus was on the new synthesis methods, new carbon sources and support materials, recently, the application possibilities are the principal arguments of the studies. The three main synthesis methods discussed in this review are the arc discharge, the laser ablation and the chemical vapour deposition (CVD) with a special regard to the latter one. In the early stage of the nanotube production the first two methods were utilized mainly for the production of SWNTs while the third one produced mainly MWNTs. The principle of CVD is the decomposition of various hydrocarbons over transition metal supported catalyst. Single-walled (SWNT), multi-walled (MWNT) and coiled carbon nanotubes are produced. In some case, interesting carbonaceous materials are formed during the synthesis process, such as bamboo-like tubes, onions, horn-like structures. In this paper, we refer to the progresses made in the field of the synthesis techniques of carbon nanotubes in the last decade.

## 1. Introduction

Carbon nanotubes were discovered by Iijima in 1991 [1]. He analyzed the samples produced by arc discharge in He atmosphere. With TEM microscopy he observed some interesting hollow tubule-like structures with nanosized diameter. Since this time these structures called carbon nanotubes made long way, but they are still in the focus of research groups dealing with different fields of science. However, the first reports about these hollow nanosized tubules were made by Russian researchers in the middle of 50’s and later on by Endo and co-workers [2,3].

Carbon nanotubes (CNTs) are characterized as a graphene sheet rolled-up to form a tube, for example a single-walled tube (SWNT). When two or more concentric tubes are placed one into another, multi-walled carbon nanotube (MWNT) is formed. Initially, the arc discharge was employed to produce carbon nanotubes. This method was known enough and utilized for the synthesis of carbon filaments and fibres. Later on other techniques such as laser ablation or chemical vapour deposition (CVD) were examined in the production of carbon nanotubes. In fact, these are the three main production methods. Some efforts were also made to look for other possibilities to grow nanotubes but they had less success. The cause may be the expensive reaction apparatus, the state or the price of the catalyst material, the strange reaction conditions, e.g., high pressure, temperatures of liquid nitrogen. So, “the old technologies” were improved, adapted to new conditions more than to discover new technologies. Today, the arc discharge and chemical vapour deposition methods are widly applied for the formation of carbon nanotubes. Many studies were made to improve either the quality or the quantity of the produced material by optimizing the synthesis process. As a result some types of CVD method were discovered such as plasma-enhanced, microwave-enhanced, radiofrequency-enhanced CVD.

During the challenge of the wonderful world of carbon nanotubes theoretical studies were also carried out. Their argument is the growth mechanism [4,5,6] of carbon nanotubes and the possibility of the formation of other nanostructures. It is supposed that the growth mechanism varies slightly from one type of production method to another. It would be nice to discover the key parameters for their formation.

Recently, the research is focused on the application possibilities of carbon nanotubes. Gratefully to their peculiar properties the field of applications is broad and it is opened from electronics, electromagnetic devices, to composite materials and optics, to biomaterials and biomedical devices. Studies in biological and pharmaceutical fields were given where carbon nanotubes can act as a part of biosensors, drug and vaccine delivery vehicles [7,8,9,10,11,12,13].

In the present review we would like to give a general overview of the different production methods of carbon nanotubes and other carbonaceous materials such as carbon onions, horns, nanospheres. The important role of the synthesis parameters which were demonstrated to be key factors during the growth process is also outlined. The results summarized in this work are essentially the studies from the last decade.

## 2. Synthesis Methods

Historically, the oldest method for the carbon nanotube production is the electric arc discharge. This technique was used already in the early sixties by R. Bacon for the synthesis of carbon fibres called whiskers. The same technique was adapted in 1990 by Krätschmer and Huffman to produce fullerenes in good yields, and later on this method was improved and applied for the synthesis of multiwall (MWNT) and singlewall (SWNT) carbon nanotubes. Other methods such as the laser evaporation/ablation and chemical vapour deposition (CVD) were also succesfully examined in the production of carbon nanotubes. The laser evaporation process is technically similar to the arc discharge method. The difference between these two methods is in the quality and purity of the obtained products. However, the arc discharge and the different types of CVD are the most promising and utilized techniques in the large scale production of carbon nanotubes and related materials.

## 3. Arc Discharge Method

**Figure 1:**
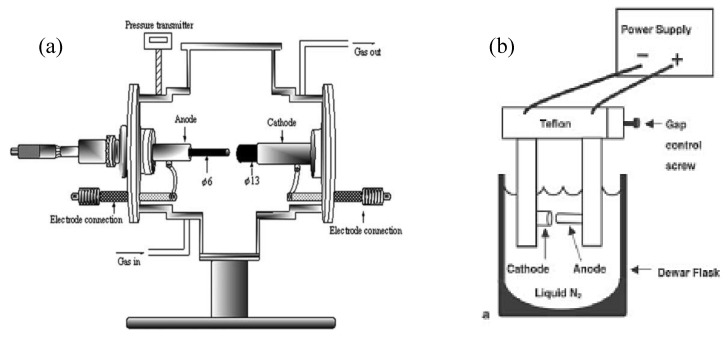
(a) Schematic representation of arc discharge apparatus. (b) Experimental arc discharge set-up in liquid N_2_.

The arc discharge technique (Figure 1 (a)) generally involves the use of two high-purity graphite electrodes. The anode is either pure graphite or contains metals. In the latter case, the metals are mixed with the graphite powder and introduced in a hole made in the anode center. The electrodes are momentarily brought into contact and an arc is struck. The synthesis is carried out at low pressure (30-130 torr or 500 torr) in controlled atmosphere composed of inert and/or reactant gas. The distance between the electrodes is reduced until the flowing of a current (50–150 A). The temperature in the inter-electrode zone is so high that carbon sublimes from the positive electrode (anode) that is consumed. A constant gap between the anode and cathode is maintained by adjusting the position of the anode. A plasma is formed between the electrodes. The plasma can be stabilized for a long reaction time by controlling the distance between the electrodes by means of the voltage (25–40 V) control. The reaction time varies from 30–60 seconds to 2–10 minutes. Various kinds of products are formed in different parts of the reactor: (1) large quantities of rubbery soot on the reactor walls; (2) web-like structures between the cathode and the chamber walls; (3) grey hard deposit at the end of cathode; and (4) spongy collaret around the cathodic deposit. The metals usually utilized were Fe, Ni, Co, Mo, Y either alone or in mixture. Better results were obtained using bimetallic catalysts. Amorphous carbon, encapsulated metal nanoparticles, polyhedral carbon are also present in the product [14,15].

When no catalyst is used, only the soot and the deposit are formed. The soot contains fullerenes while MWNTs together with graphite carbon nanoparticles are found in the carbon deposit. The inner diameter of the MWNTs varies from 1 to 3 nm, the outer diameter varies in the range of 2–25 nm, the tube length does not exceed 1 μm, and the tubes have closed tips.

When metal catalysts are co-evaporated with carbon in the DC arc discharge, the core of the deposit contains MWNTs, metal filled MWNTs (FMWNTs), graphitic carbon nanoparticles(GNP), metal filled graphite carbon nanoparticles (FGNP) and metal nanoparticles (MNP), while the powder-like or spongy soot contains MWNTs, FMWNTs and SWNTs. The SWNTs have closed tips, are free of catalyst and are either isolated or in bundles. Most of the SWNTs have diameters of 1.1–1.4 nm and are several microns long. The collarette is mainly constituted of SWNTs (80%), isolated or in bundles, but it is only formed in the presence of certain catalysts.

The physical and chemical factors influencing the arc discharge process are the carbon vapour concentration, the carbon vapour dispersion in inert gas, the temperature in the reactor, the composition of catalyst, the addition of promoters and the presence of hydrogen. These factors affect the nucleation and the growth of the nanotubes, their inner and outer diameters and the type of nanotubes (SWNTs, MWNTs). The amount of carbon nanoparticles was found to diminish when pure hydrogen was used during the reaction [14].

Let us emphasize that Journet *et al*. [15] have proposed a large scale production of SWNTs in gram quantities using electric-arc technique. The catalysts used were Ni-Co, Co-Y and Ni-Y in various atomic percentages.

In Table 1, the studies reporting the kind pf produced materials and the variation of synthesis parameters are listed.

**Table 1:** Carbonaceous nanomaterials synthesized by arc discharge method applying different synthesis conditions.

Method	Product	Comments	Conditions	References
Plasma rotating	CNTs	Large scale	Pure graphite electrodes, He	[37]
Arc discharge	CNTs		Deionized water	[32]
	MWNTs, carbon onions			[31]
	CNTs	Metal filled		[30]
	MWNTs, SWNTs, carbon nanocapsules		NaCl solution	[29]
	MWNTs, multishell carbon onions	Distorted morphology, irregular shape	Liquid environments	[28]
Arc discharge	SWNTs, nanohorns		Liquid N_2_	[27]
	Spheroidal nanocarbons, graphite sheets, tube-like nanocarbons		Toluene, different types of catalysts	[25]
Arc discharge	CNTs	Continuous production		[26]
	SWNTs, fullerenes, metallofullerenes	d = 0.9–1.4 nm	Y/Ni and CaC_2_/Ni catalyst, He	[23]
	SWNT fibers	High purity		[19]
	SWNTs, CNT ribbons	High yield	Ho/Ni catalyst	[18]
	MWNTs, sheet like structures, spherical particles, beaded CNTs	The product type depends on the catalyst composition	PVA, PVA/Fe catalysts, various Fe sources	[17]
Arc discharge	DWNTs	Large quantity, high quality, d = 2–6 nm	KCl/FeS catalyst, H_2_	[24]
			Mixture of Ni/Co/Fe small amount of S, Ar:H_2_	[20]
			FeS, CoS, NiS catalysts, H_2_	[21]
		Bundles of high quality		[22]
	MWNTs	Optimization process	Graphite electrodes, H_2_	[35]
Pulsed arc	CNTs, onion-like particles	Straight with d = 20 nm, aggregations of nano-onions d = 15–20 nm	Pure graphite rods, deionized water	[36]
AC-Arc discharge	MWNTs, nano-onions	Well graphitized, closed ends, nano-onions d = 20–50 nm	Deionized water, various carbon sources and catalysts	[33,34]

### 3.1. Catalyst composition and carbon sources

**Figure 2:**
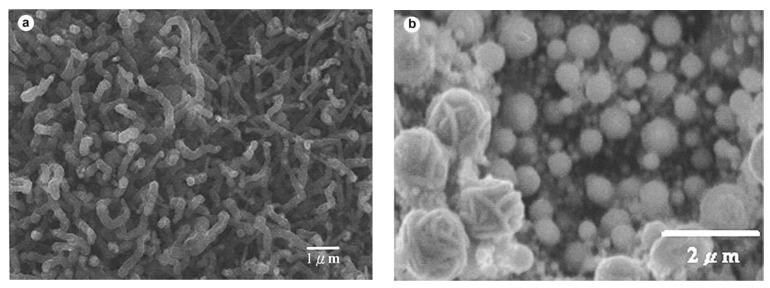
Effect of PVA/Fe powder catalyst on the formation of MWNTs. (a) MWNTs formed by PVA:Fe 9/1 fillings; (b) spherical-like carbon particles formed by PVA:Fe 1/9. Ref [17].

Wang *et al*. [17] utilized polyvinylalcohol (PVA) and PVA/Fe mixture as catalyst. They found that the type of the product varies with the PVA/Fe ratio. When high concentration of PVA was used in the catalyst mixture high density of entangled MWNTs of diameters at about 60–190 nm were obtained (Figure 2 (a)). Oppositely, using high concentration of Fe spherical particles (Figure 2 (b)) were synthesized and no MWNTs formation was observed. When only PVA was used MWNTs with diameters in the range of 30–60 nm, sheet-like carbon structures and beaded carbon nanotubes were found. The authors suppose that the latter ones were formed because of the current variation during the synthesis. It is known that different rare-earth elements have strong influence on the quantity and the nanostructure of the SWNTs produced, e.g., Eu/Ni catalyst produced very few SWNTs with nanoparticles, while applying Ce/Ni catalyst small diameter SWNTs were obtained. However, Ni has an essential role in the synthesis of SWNTs by arc discharge method and the rare-earth metals play the role of the co-catalyst. Yao *et al*. [18] tested a Ho/Ni catalyst with a slightly modified conventional arc-discharge apparatus. The product contained web- and collar-like assemblies of SWNTs, SWNT ribbons with length up to 10–20 cm and bundle diameters at about 10–30 nm. From Raman measurements the diameter of the individual SWNTs was established to be in the range of 1.30–1.64 nm.

**Figure 3:**
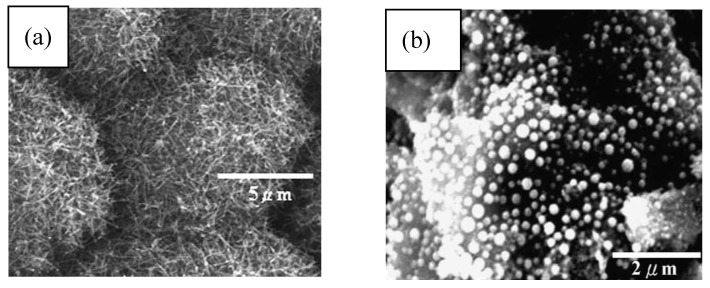
Effect of PVA/Fe_2_(SO_4_)_3_ on the formation of MWNTs. (a) MWNTs formed by PVA:Fe_2_(SO_4_)_3_ 9/1. (b) Spherical nanoparticles formed by PVA:Fe_2_(SO_4_)_3_ 1/9. Ref [17].

**Figure 4:**
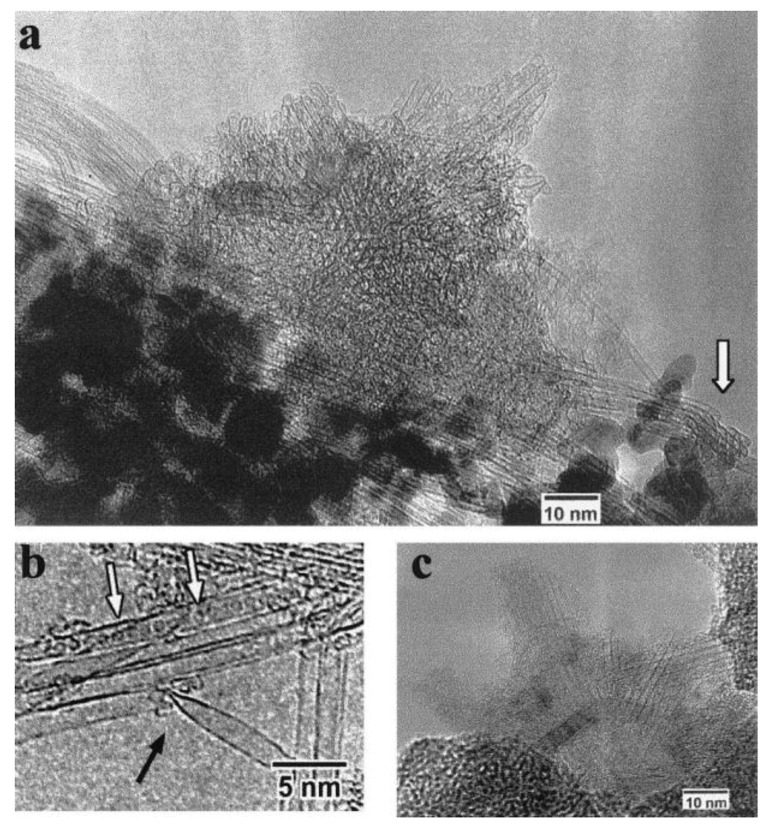
HRTEM images of SWNTs produced using Y/Ni catalyst. (a) A flower-like SWCNTs. (b) Individually grown SWCNTs with a diameter as large as 1.6 nm (a kink is indicated by a bold arrow). (c) A radial SWCNT bundles with a carbon nanoparticle core. Encapsulation of C_60_ molecules (indicated by open arrows) in a SWCNT is shown in (a, b). Ref [23].

**Figure 5:**
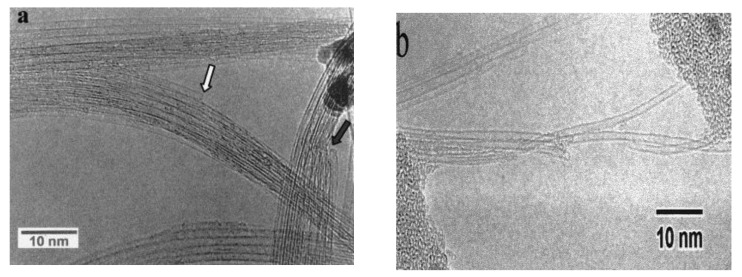
HRTEM images of SWCNTs produced with (a) Y/Ni and (b) CaC_2_/Ni catalyst. Ref [23].

**Figure 6:**
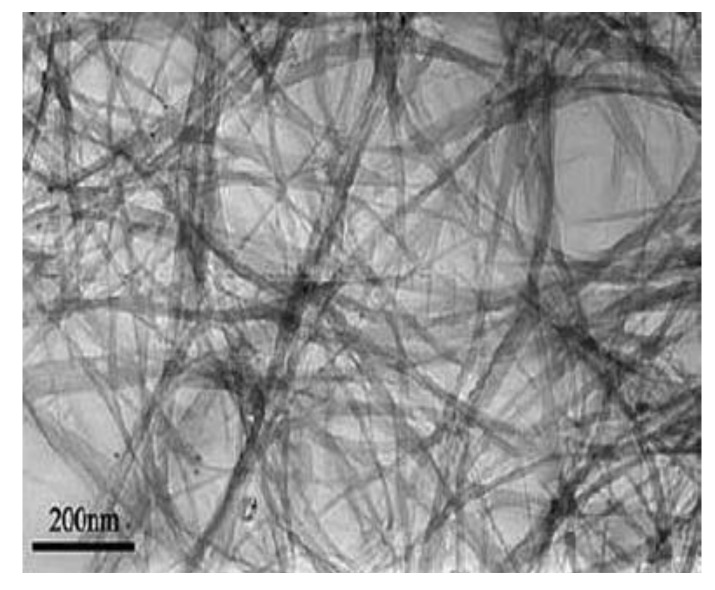
TEM image of purified DWNT bundles obtained on KCl/FeS catalyst. Ref [24].

Gu *et al*. [19] referred to high purity SWNT fibers utilizing Y/Ni catalyst with a small amount of sulphur. Also DWNTs were prepared using a sulphur containing catalyst or by adding sulphur in small quantities into the reaction environment [20,21,22]. What is the role of sulphur? In the literature, there are contradictory contributes on the role of sulphur in the growth mechanism. As it was observed by Wang *et al*. [17], the addition of a small amount of sulphur can lead to the formation of filled CNTs, or can increase the MWNTs diameter using PVA/Fe catalyst with Fe(SO_4_)_3_ as source of sulphur (Figure 3). The graphite electrodes filled with Y-Ni alloy and Ca_2_C-Ni powder were tested in the synthesis of SWNTs [23]. High density of SWNT bundles with diameter of 20–30 nm and length about 15 µm, some individual SWNTs, and encapsulated C_60_ molecules were found in the soot (Figure 4 and Figure 5). The diameter of individual SWNTs in the bundles was established either from HRTEM images or from Raman measurements, and it was found to be in the range of 1.3–1.4 nm for the Y-Ni catalyst and 0.9–1.1 nm for the Ca_2_C-Ni catalyst. However, the Y-Ni catalyst was confirmed as the most effective catalyst for the high scale production of SWNTs in the arc process (carbon yield at about 60%). Qiu *et al*. [24] tried KCl with FeS as catalyst. The obtained products were deposited either on the substrate as thread-like soot containing bundles of DWNTs, or on the inner wall of the reaction chamber as cloth-like soot containing individual carbon nanotubes (Figure 6). From the study of the series of experiments with various KCl contents it was found that the CNT formation increased by increasing the amount of KCl but it had an upper limit of 3 wt %. The KCl was replaced by other chemicals containing potassium or chloride to examine the role of these elements. The authors supposed that chloride can act as promoter in the formation of carbon fragments containing six- or five-membered rings which could be identified as the initial fragments’caps or nuclei.

Toluene was utilized as hydrocarbon source varying the element of the electrodes from pure graphite, to Mo, Fe, and Ni rods in the experiments of Okada’s group [25]. No significant relationship was observed between nanocarbon formation and the conditions of the discharge process. The purity and crystallinity of the products increased in the following sequences: graphite – only spheroidally shaped structures with low crystallinity; Mo—spheroid nanocarbons; Fe—graphite layers; Ni—graphite sheets, tube like structures with several graphite sheets.

### 3.2. The reaction environments

As reaction environments liquid N_2_ (Figure 1(b)), deionized water and other aqueous solutions were examined. Liquid N_2_ was utilized for the first time by Ishigami *et al*. [26]. They obtained MWNTs. Sano *et al*. [27] referred to the preparation of SWNTs using Ni composite anode in liquid N_2_. Antisari and co-workers [28] reported a comparison between liquid N_2_ and deionized water. When liquid N_2_ was used the kind of the synthesized products depended on the applied voltage. Amorphous carbon and a few CNTs were obtained applying lower voltage than 22 V, while a microstructure similar to the nanoporous carbon was produced at higher voltage than 27 V. On the other hand, in the range of 22 V–27 V high density of MWNTs of distorted morphology and degraded structure, and irregularly shaped multishell carbon onions were observed in the product. In the case of the deionized water a voltage of 28 V was applied and MWNTs embedded in amorphous carbon matrix were produced. The authors favored deionized water as reaction environment for the production of carbon nanotubes because the quality of the nanotubes produced in liquid N_2_ was good only at the top surface of the sample. In the lower areas some degradation mechanisms occurred which can be related to the fast and violent evaporation operating in liquid N_2_. Despite the extremely low temperature of liquid nitrogen, the strong evaporation resulting from the operation of the arc discharge does not allow good thermal exchange between the synthesized material and its surroundings. Therefore, liquid nitrogen provides less efficient cooling than deionized water does, and the MWCNTs produced exhibit distorted morphology and degraded structure. Consequently, another adequate liquid media must be found which has remarkable cooling ability but does not vaporize strongly during arc discharge. The NaCl solution could be a good medium because its cooling ability significantly exceeds that of deionized water. A 0.25 M NaCl solution was used in combination of 5 at % Fe powder graphite electrode [29] to produce carbon nanotubes. The product deposited on the bottom of reaction vessel contained MWNTs, carbon nanocapsules, a few SWNTs with diameters of about 0.9 nm, and cubic structures of Fe_2_O_3_. Deionized water was applied in the studies of Hsin *et al*. [30] and Lange *et al*. [31]. In the first case metal filled MWNTs, in the latter case carbon onions and MWNTs were observed in the synthesis products. Zhu and co-workers [32] synthesized MWNTs in deionized water and aqueous solutions of NiSO_4_, CoSO_4_ and FeSO_4_.

The AC arc experiments [33,34] were performed between two graphite electrodes submerged in deionized water in a glass vessel. The angle between the electrodes was varied between 30° and 180° to determine its effect on the product yield. Different aromatic hydrocarbons such as toluene, xylene, cyclohexane, cyclohexanone, n-hexane, n-heptane, n-octane and n-pentane were used as carbon feedstock, and ferrocene, cobaltocene and nickelocene as catalyst source. The discharge current was set to 40 A with a discharge voltage of 25 V, and it was possible to keep the apparatus running for 1–2 h continuously. All the compounds tested were found to be suitable for nanotube production. The highest yield and the best quality were obtained when a mixture of ferrocene–nickelocene was used as catalyst and xylene as carbon source. The highest nanotube yield and lowest amount of impurities were found at 90° electrode angle. TEM investigations showed that the MWCNTs were always agglomerated with carbon nano-onions. These agglomerations of MWCNTs and bucky onions with dimensions ranging from a few hundred nanometers to several microns are bonded together chemically. This is presumably due to the presence of extra atomic oxygen and hydrogen in the arc plasma compared to the conventional arc growth technique. The MWCNTs are well graphitized, closed at the ends with typical outer diameters of 10–35 nm and length of a few microns. However, the outer 1–2 walls of these tubes are often damaged and they are partly covered with thin disordered material. This can also be the consequence of the reactive environment. Most of the bucky onions are nearly spherical with more or less polyhedral character; their typical dimensions are in the range of 20–50 nm.

### 3.3. The pressure of gases

The type of the produced material is demonstrated to be highly influenced by the applied pressure of the carrier and/or inert gas. Shi *et al*. [23] observed that at their reaction conditions the pressure of He of 700 Torr gives high density of SWNT bundles with d = 20–30 nm and length up to 15 microns. In contrast, applying a He pressure lower than 300 Torr the typical product was a mixture of fullerenes and metallofullerenes. Qiu and coworkers [24] found that the formation process of DWNTs was highly sensitive to the composition of the catalyst and H_2_ pressure. Utilizing 3 wt % KCl/graphite catalyst the optimal pressure of H_2_ was found to be 350 Torr. This pressure favored the growth of DWNTs in high yield. Some tentatives to optimize the arc discharge production of MWNTs was also reported [35]. The effect of the current density and H_2_ pressure variation was studied. It was observed that the yield and the purity of the obtained material increased increasing these parameters. The best result was found applying 190 A/cm^2^ current density and 500 Torr of H_2_.

### 3.4. Types of arc discharge methods

Imasaka *et al*. [36] reported on the intermittent arc discharge process in water producing carbon nano-onions and nanotubes. This technique permits several millisecond pulse duration which is much longer than that of the pulsed arc method with microsecond pulse duration. The product obtained was either a floating powder containing uniformly dispersed fine spherical particles, or a sediment composed of straight MWNTs with length in the range of 100–500 nm and aggregated onion-like nanoparticles.

Lee *et al*. [37] synthesized CNTs by plasma rotating arc discharge process. They observed that the nanotube yield was increased as the rotation speed of the anode was increased and the collector became closer to the plasma.

A remarkable study has been carried out very recently on the mechanism of formation of SWNT from acetylene on Ni nanoparticles using field emission microscopy (FEM) [38]. It was shown that the CNTs often rotate axially during growth following the screw-dislocation-like (SDL) model and this rotation is driven by the insertion of dimers. This scheme implies that there must be both longitudinal and rotational sliding between the nanoparticle and the edge of the CNT in order to give space to the newly accreted carbon dimers.

## 4. Laser Ablation Method

**Figure 7:**
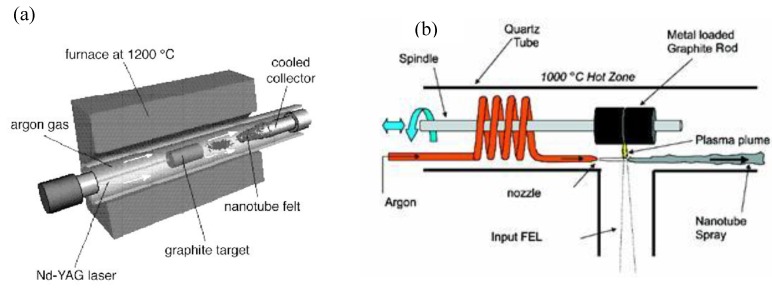
Schematic synthesis apparatus. (a) Classical laser ablation technique. (b) Ultrafast laser evaporation (FEL-free electron laser).

The pulsed laser-ablation process for the production of single-wall carbon nanotubes was developed by Guo *et al*. [39] at Rice University. The method they used is described below. In 1996, Smalley *et al*. successfully developed a laser ablation method for the “mass production” of SWNTs [40]. Other improvements were made by Thess *et al*. [41] and Rao *et al*. [42] using double beam laser. Nanotubes produced by laser ablation have higher purity (up to about 90% pure) and their structure is better graphitized than those produced in the arc process. The disadvantage of this method is the small carbon deposit. The laser ablation technique favors the growth of SWNTs. MWNTs are generated only employing special reaction conditions. Figure 7 (a) represents a schematic classical set-up of this method.

The oven laser-vaporization apparatus used by Guo’s group [39] was the same as the one used to produce fullerenes, metallofullerenes, and multiwalled nanotubes [43]. A laser beam (532 nm), was focused onto a metal-graphite composite target which was placed in a high-temperature furnace (1200 °C). The laser beam scans across the target surface under computer control to maintain a smooth, uniform face for vaporization. The soot produced by the laser vaporization was swept by the flowing Ar gas from the high-temperature zone, and deposited onto a water-cooled copper collector positioned downstream, just outside the furnace. The targets were uniformly mixed composite rods made by the following three-step procedure: (i) a paste produced from mixing high-purity metal or metal-oxide with graphite powder and carbon cement at room temperature was placed in a mold; (ii) the mold was placed in a hydraulic press equipped with heating plates and baked at 130 °C for 4–5 h under constant pressure; (iii) the baked rod was then cured at 810 °C for 8 h under Ar flow. Fresh targets were heated at 1200 °C under flowing Ar for 12 h. Subsequent runs with the same target proceeded after two additional hours heating at 1200 °C. The following metals were used: Co, Cu, Nb, Ni, Pt, Co/Ni, Co/Pt, Co/Cu, Ni/Pt. A composite ablation target consisting of 1 at % each of Ni and Co uniformly mixed with graphite resulted in the best purity and yield of SWNTs of the configurations tested. The major impurities in the product are amorphous carbon, graphite particles, catalysts, and fullerenes. Other impurities that have been detected include silicon and various hydrocarbons, which are probably due to impurities introduced from unknown sources [39].

The studies realized until today showed that the quantity and the quality of produced material can be controlled to some extent by changing: (i) the type of metal catalysts and their ratio [44,45,46]; (ii) the kind of the ambient gas and its pressure [47,48,49]; (iii) the temperature of the reaction furnace [50,51,52]; and also (iv) the laser parameters [53,54,55]. Below we report some comments how the variation of the reaction parameters can effect the quality, structure, and quantity of the obtained materials.

### 4.1. Target composition

Maser *et al*. [56,57,58] carried out a more complex work in which they studied the effect of metal concentration in the catalyst, gas flow and pressure on the growth of SWNTs. Isolated SWNTs were formed using monometallic Co or Ni/graphite target. High amounts of SWNT bundles were obtained applying bimetallic graphite catalyst Ni/Y with the concentration of Ni always higher than that of Y, or Ni/Co in equal concentration (2/2 at %). No differences were found in the sample quality using Ar or N_2_. In both cases high yields of SWNTs were obtained at pressures in the range of 200–400 Torr. No carbon nanotubes were formed at pressures below 100 Torr. Employing He as inert gas no CNT formation was observed. Changing the gas flow rates does not lead to significant changes in the quantity and the quality of the produced CNTs. Great differences were observed employing continuous or pulsed laser mode. While in the first case high quality SWNTs were produced with an evaporation rate of 200 mg/h, and lowering the power density no significant changes in the sample quality was observed, in the latter case the main product was amorphous carbon with an evaporation rate of 4 mg/h, and no heating of the target was observed.

Bolshakov and collaborators [59] substituted the solid target by a gas-powder suspension composed by 2.5 at % Co and 2.5 at % Ni. This experiment was made in effort to decrease the thermal conductive losses of laser power to reach more effective utilization of the adsorbed laser radiation for material evaporation. Besides, this laser–powder technique does not require the production and exchange of bulk solid targets which makes the synthesis process continuous. Raman spectroscopy and HRTEM investigations of the obtained deposits have revealed a SWNT abundance of 20–40%. The diameter of produced SWNTs was around 1.2–1.3 nm.

### 4.2. Carrier gas parameters

Generally, Ar and/or H_2_ are used as carrier gas in the laser ablation process. It is well known that changing any conditions of ambient gas influences the product quantity and quality. In the following some works are described in which the ambient gases were different from Ar and H_2_.

**Figure 8:**
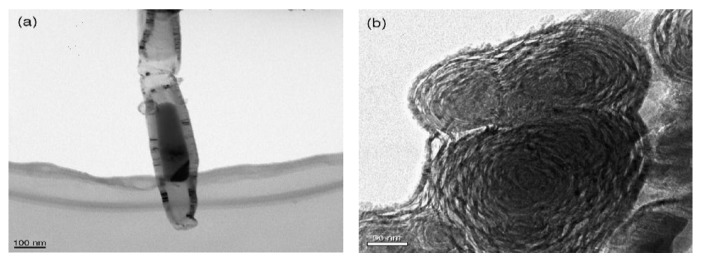
TEM images of carbon nanostructures obtained on Ni/Co (0.5/0.5 at %) catalyst in O_2_ atmosphere. (a) Nanotube with metal encapsulation and large central channel. (b) Carbon nano-onions. Ref [60].

O_2_ and Ar were applied as carrier gas using pure graphite and graphite/Ni/Co target for the synthesis of carbon nanotubes by KrF excimer laser ablation [60]. Amorphous carbon was formed with pure graphite either in O_2_ or Ar ambient. Changing the composition of the target to C/Ni or C/Ni/Co CNTs and carbon onions (Figure 8) were produced in O_2_ atmosphere while only amorphous carbon was deposited in Ar atmosphere. The formed carbon nanotubes have bamboo-like structure with large internal (50–100 nm) and also external (100–200 nm) diameters. These carbon nanotubes have much larger diameter than those generally produced by laser ablation (for MWNTs d = 10–40 nm). The carbon onions with diameter of 100–200 nm were observed either individually or in clusters. The authors believe that the O_2_ molecules play a key role in the reactions leading to the CNTs formation. Spectral modelling shows that the vibrational-rotational temperatures for C_2_ produced in O_2_ remain around 5000 K for nearly 20 microseconds, but drop rapidly in Ar. An exothermic reaction with O_2_ producing 4 eV of energy can explain these observations.

Muñoz *et al*. [61] performed the synthesis on C/Ni/Y target in Ar, N_2_ and He atmosphere at 50–500 Torr of pressure. The authors observed the formation of high yield filamentous soot with web-like texture applying 200–500 Torr of pressure. The diameter of SWNT bundles observed in this material was about 10–20 nm and their length was more than 1 µm in Ar and N_2_ atmosphere. In contrast, using He no significant amount of SWNTs and filaments was observed in the soot. The influence of the nature of the gas and its pressure on the formation of SWNTs can be explained by correlating these parameters directly to the change of the temperature and concentration of the species contained in the vapor plume and the temperature gradient. Increasing the pressure will result in a too high collision probability of the evaporated species with the ambient gas molecules resulting therefore in an increased cooling rate and subsequently the SWNT formation efficiency reduces. Lighter gases are more efficient for rapid cooling than heavier ones, but the temperature gradient (cooling rate) becomes too large for the SWNTs formation.

**Figure 9:**
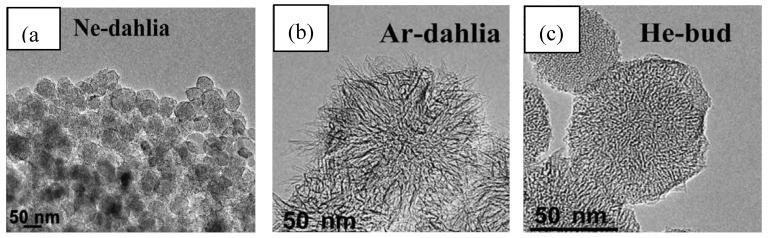
TEM images of carbon nanostructures synthesized on pure graphite target. (a) Ne atmosphere-dahlia type. (b) Ar-dahlia type. (c) He-bud type. Ref [62].

Azami *et al*. [62] reported carbon nanohorn formation by CO_2_ laser ablation of pure graphite. They studied the effect of Ar, Ne and He gas on the structure of the obtained products. Applying Ar and Ne the quality of the synthesized material does not vary a lot. Dahlia-type carbon nanohorn aggregates were obtained (Figure 9 (a, b)). Noticeable difference was observed in the size of these aggregates. Using Ar, nanohorns with diameter of 100 nm, while in the presence of Ne smaller aggregates of about 50 nm were formed. Utilizing He as ambient gas bud-type nanohorns with an average diameter of 70 nm were produced (Figure 9 (c)).

### 4.3. The effect of laser parameters and furnace temperature

**Figure 10:**
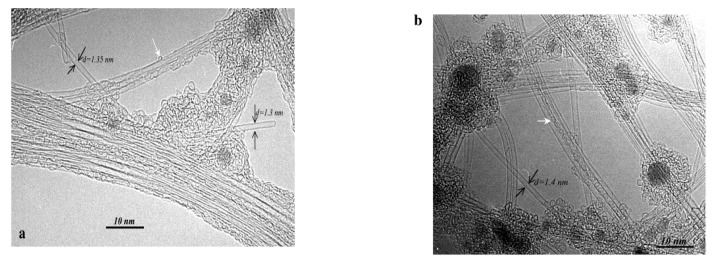
HRTEM photos of SWNTs produced with (a) Ni/Y catalysts and (b) Ni/Co catalysts. Ref [63].

In addition to the commonly used Nd:YAG lasers for SWNTs synthesis, CO_2_ lasers (either pulsed or continuous wave) can also produce SWNTs even at room temperature [51,52,56]. Their longer wavelength heats up the target surface acting thus as a local furnace. At room temperature, the continuous-wave mode of the CO_2_ lasers have been reported to be more effective in the production of a higher SWNT yield than in the pulsed regime. It was shown that different laser parameters have also important influence on the structure of synthesized materials. Eklund and co-workers [63] first used the ultrafast (subpicosecond) laser pulses for the large-scale production of single-walled carbon nanotubes (SWNTs) by pulsed laser vaporization (PLV) technique. The SWNTs thus prepared showed remarkable uniformity in diameter, which is mostly distributed around 1.3 nm (Figure 10). Furthermore, the SWNTs appear to be entangled threads with lengths of several hundred micrometers. Each thread is a bundle of SWNTs with hexagonal close packing. The bundle formation was more dominant with the laser method than with the arc method.

Continuous CO_2_ laser was applied to vaporize the C/Ni/Co (0.6 at %) target in Ar atmosphere with laser power of 400–900 W [55]. It was found that lower power than 400 W is not sufficient to vaporize the target. The average diameter of SWNTs increases slightly with increasing laser power. Bamboo-like CNTs were observed at a power of 500 W. Applying higher laser power SWNT bundles with diameter of 6–20 nm were formed, that shows the importance of power applied.

The effect of laser intensity and furnace temperature on diameter distribution, yield and physical characteristics of SWNTs was also studied [50]. Pulsed Nd:YAG laser green pulse with intensity of 0.5–4.6 × 10^9^ W/m^2^; infrared pulse with intensity of 0.6–5.9 × 10^9^ W/m^2^, and double pulse with intensity of 0.6–5.6 × 10^9^ W/m^2^ were tested. The furnace temperature was varied in the range of 800–1150 °C applying a composite target in Ar atmosphere. The yield and structural characteristics of the produced material were highly dependent on the laser intensity which was proved by Raman analysis and HRTEM observations. The higher intensity and high furnace temperature favors the formation of larger SWNTs with diameter at about 1.4 nm. The bundle diameter increases with the laser intensities up to a maximum value after which it decreases even if the laser intensity is further increased. The largest bundles are formed at the optimal laser intensities, in this case at 2.3 × 10^9^ W/m^2^ when entangled SWNT bundles long 10–20 micron and some nanoparticles were deposited. The rope diameter of SWNTs produced by Nd:YAG laser linearly increased with the furnace temperature: 2–13 nm for 750–1150 °C. The optimal laser intensity regardless of laser pulse configuration, corresponds to an optimal heating of the target surface that favors the growth of SWNTs and their arrangement in large bundles. At the optimal laser intensity it is believed to obtain: the highest SWNT yield, the largest SWNT bundles diameter, the lowest level of amorphous carbon and/or disordered sp^2^ carbon in the deposit.

A comparison study of the Nd:YAG with CO_2_ laser was done by Yudasaka *et al*. [64]. They found that a longer wavelength combined with a longer pulse width leads to a higher yield. There are studies on the influence of pulsed and continuous laser mode. It was demonstrated that the continuous mode is more efficient for the high yield production of CNTs.

Kusaba and Tsunawaki [65] produced SWNTs using XeCl excimer laser ablation technique changing the process temperature from 1000 °C to 1350 °C. The best results have been found at the T = 1000 °C in the form of web-like deposit. The SWNT bundles of d = 20 nm were found by TEM observations. Raman analysis demonstrated that most of the individual SWNTs have a diameter in the range of 1.2–1.7 nm. Entangled and cross-linked nanotubes were present in the samples.

Braidy *et al*. [66] performed experiments using UV laser KrF excimer to test the effect of furnace temperature (25–1150 °C) on the quality of produced materials. They observed the formation of thicker SWNT bundles in higher yield increasing the process temperature. The same experiments were carried out applying a repetition rate of 150 Hz of laser. In this case, entangled and large bundles of SWNTs with average diameter of 70 nm were obtained at the furnace temperature of 1150 °C, which was four times higher than that produced using 30 Hz of repetition rate. The product was free of amorphous carbon. The possible explanation could be that under high repetition rate conditions the local temperature increases on the target surface resulting in a great heat accumulation favoring the growth of large catalytic particles, and therefore large bundles of nanotubes.

Table 2 reports the synthesis of carbonaceous materials by laser evaporation method using various modes of laser beam under different conditions.

**Table 2:** Carbon nanstructures synthesized by laser evaporation at different reaction conditions.

Method	Product	Conditions	References
XeCl excimer	SWNT bundles, fullerenes	Process temperature: 1000–1350 °C; C/Ni/Co; Ar	[65]
KrF excimer	MWNTs, nano-onions	Target composition: C/Ni, C/Ni/Co; gas nature: Ar, O_2_; room T	[60]
CO_2_ continuous wave	SWNT bundles, bamboo-like structures	Laser power: 400–900 W; C/Ni/Co, room T Ar: 200–400 Torr;	[55]
Pulsed Nd:YAG	SWNT bundles	Laser intensity: 532 nm, 1064 nm, double beam 532 and 1064 nm; C/Ni/Co; Ar; Furnace T: 800–1150 °C	[50]
CO_2_ continuous wave		Gas-powder suspension catalyst; Ar, N_2_; 1100 °C	[59]
		Gas nature Ar, He, N_2_ 50–500 Torr; C/Ni/Y	[61]
CO_2_ continuous and pulsed wave	SWNTs	Catalyst composition, gas conditions: Ar, He, N_2_ 50–500 Torr, laser power density: 12–9-6 kW/cm^2^	[58]
		Configuration of laser wave	[64]
		Effect of the growth temperature	[52]
Pulsed Nd:YAG laser	Thin SWNTs	Target composition, reaction T and gas flow velocity	[46]
CO_2_ pulsed laser	SWNTs	Target composition	[56]
		Gas nature and its pressure	[57]
CO_2_ continuous wave	CNTs	Effect of furnace temperature	[51]
Pulsed double beam Nd:YAG	SWNTs	Effect of the laser intensity	[41]
Pulsed Nd:YAG			[42]
KrF excimer		Furnace T = 550 °C	[49]
Pulsed Nd:YAG		Laser parameters	[53,54]
		Target composition	[44,45]
Pulsed double beam Nd:YAG		Gas pressure, flow	[47,48]
KrF excimer UV laser	SWNT bundles	Furnace temperature 25–1150 °C; Ar, C/Ni/Co	[66]
CO_2_ laser	Carbon nanohorns	Gas nature Ar, Ne, He; Pure C	[62]

## 5. Chemical Vapour Deposition Method

While the arc discharge method is capable of producing large quantities of unpurified nanotubes, significant effort is being directed towards production processes that offer more controllable routes to the nanotube synthesis. A class of processes that seems to offer the best chance to obtain a controllable process for the selective production of nanotubes with predefined properties is chemical vapour deposition (CVD) [67]. The formation of carbon filaments from the catalytic decomposition of carbon-containing gas over metal surfaces has been known for a long time [2,3,68]. However, there was no evidence that this technique could be used to synthesize carbon nanotubes until Yacamàn *et al*. [69] succeeded. Today, the catalytic chemical vapour deposition (CCVD) method is considered as the only economically viable process for large-scale CNT production and the integration of CNTs into various devices [70] as it is shown by the large-scale production of quite pure carbon nanotubes by NANOCYL founded by one of the authors [71].

**Figure 11:**
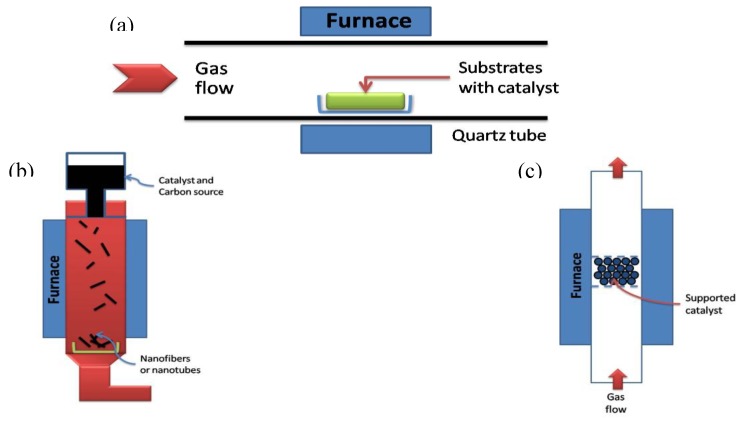
Schematic demonstration of CVD method. (a) Horizontal furnace. (b) Vertical furnace. (c) Fluidized bed reactor.

In principle, chemical vapour deposition is the catalytic decomposition of hydrocarbon or carbon monoxide feedstock with the aid of supported transition metal catalysts. Generally, the experiment is carried out in a flow furnace at atmospheric pressure. There are two types of furnace modality, one is a horizontal configuration, the second is a vertical configuration (Figure 11). The application of horizontal furnace is the most popular. Here the catalyst is placed in a ceramic or quartz boat which is put into a quartz tube. The reaction mixture containing a source of hydrocarbon and an inert gas is passed over the catalyst bed at temperatures ranging from 500 °C to 1100 °C. The system is then cooled down to room temperature. The vertical furnace configuration is usually employed for the continuous mass production of carbon fibers/nanotubes. The catalyst and carbon source is injected at the top of the furnace and the resultant filaments grow during flight and are collected at the bottom of the chamber. Ultrafine metal catalyst particles are either introduced into the reactor directly or formed *in situ* using precursors such as metallocenes. The fluidized bed reactor is a variation of the vertical furnace. Supported catalysts are usually placed in the center of the furnace and an upward flow of carbon feedstock gases is used. The fluidization process involves the supported catalysts to remain much longer in the furnace than in the vertical floating tehnique [68]. The general nanotube growth mechanism in the CVD process involves the dissociation of hydrocarbon molecules catalyzed by the transition metal, and the saturation of carbon atoms in the metal nanoparticle. The precipitation of carbon from the metal particle leads to the formation of tubular carbon solids in a sp^2^ structure. The characteristics of the carbon nanotubes produced by CVD method depend on the working conditions such as the temperature and the operation pressure, the kind, volume and concentration of hydrocarbon, the nature, size and the pretreatment of metallic catalyst, the nature of the support and the reaction time [8]. By varying the active particles on the surface of the catalyst, the nanotube diameter can be controlled. The length of the tubes depends on the reaction time; even up to 60 mm long tubes can be produced [72].

Using CVD method, several structural forms of carbon are formed such as amorphous carbon layers on the surface of the catalyst, filaments of amorphous carbon, graphite layers covering metal particles, SWNTs and MWNTs made from well-crystallized graphite layers. This method allows selective CNT growth in a variety of forms, such as powder [73,74] and aligned forrest of CNTs [75,76]. The produced tubes can adopt various shapes; they can be straight, curved, planar-spiral, and helix, often with a remarkably constant pitch. The fibers often have an amorphous carbon coating, and metal particles are sometimes found at their tips. In particular, CCVD provides the possibility of growing CNTs from controlled surface sites by catalyst patterning on a desired substrate [77,78]. This permits specific applications, e.g., field-emission displays [79], specific architecture of a nanotube device [80] or probe tips of scanning probe microscopes (SPM) [81,82]. Planar arrays of aligned nanotubes over large areas with sufficiently high density and order have been also grown on single-crystal substrates of sapphire or quartz, which for instance enable their easy integration into high-performance planar devices [83,84,85]. 

In Table 3, the formation of various kinds of carbonaceous materials is listed regarding to the different CVD methods and different reaction conditions.

**Table 3:** Summary of the articles reporting CVD production of carbon nanoarchitectures.

Method	Product	Characteristics	C source, catalyst	References
Water-assisted	Vertically aligned SWNTs, DWNTs	High yield	With buffer layer	[128]
O_2_-assisted plasma-enhanced				[129]
Microwave plasma-enhanced				[130]
Microwave plasma-enhanced	Verticaly aligned SWNTs		Co-Ti/Si substrate without a buffer layer	[131]
Simple CVD	Carbon spheres	Ball-like, chain-like morphology	Toluene, without catalyst	[144]
	Filled CNTs, Fe_3_C nanowires		Acetylene, titanate modified palygorskite	[165]
	Regularly coiled carbon fibers	Large scale	Acetylene, small amount of S or P	[148]
Hot wire CVD	3D double-helix microcoils	Amorphous structure	Methane, Ni catalyst	[147]
DC-PECVD	Carbon fibres			[146]
Microwave plasma-enhanced	Carbon nanofibres	Vertically aligned	Catalyst free, CO/Ar/O_2_, low temperature	[145]
Hot filament-enhanced	SWNTs, MWNTs	Perpendicularly or vertically aligned	Fe-Co/SiO_2_ with or without of Si support	[127]
Alcohol CVD	CNTs	Multibranched morphology	Cu/MgO	[142,143]
High power laser pulse alcohol CVD	SWNTs		Solid metal target	[135]
Alcohol CVD		High purity	Ferrocene-ethanol	[89,132,133,134]
Thermal CVD	CNTs	Aligned	Co/SiO_2_, Ar/H_2_ and NH_3_/N_2_	[140,141]
Simple CVD	SWNTs	Individual and in bundles	Fe-Mo/Si substrate, methane	[139]
Microwave plasma-enhanced	CNTs	Well aligned, curved with random orientation	Fe/sapphire, Ni-Fe/glass, Cr-Fe/glass, Fe/Si, stainless steal	[126]
Injection CVD	MWNT films	Aligned, encapsulated nanoparticles	Quartz substrate, ferrocene/toluene	[125]
	MWNTs	Mainly straight, some thick CNTs	Different carbon sources and metal catalyst	[33]
CVD	CNTs, carbon onions		Ni/Al	[120,121]
		Metal filled, bamboo shaped	Ni/Cu/Al, methane	[122,123]
	MWNTs		K-doped Co and Co-Fe/zeolite and CaCO_3_	[114]
	SWNTs, DWNTs		Fe-Mo/MgO	[110,111,112,113]
	CNTs		Different metals and rare-earth promoters	[109]
CVD	Aligned CNTs		Single-crystal of sapphire or quartz	[83,84,85]
	CNTs, graphite layers, filaments		Different types of catalysts	[73,74,75,76]
	Helicoidal CNTs	Regular and irregular shape		[152,159,160]
Alcohol CCVD	CNTs	Various morphology depending on the metal film thickness	Co/Si, Co-Mo/Si, Co/quartz, Co-Mo/quartz	[136,137]
Ultrasonic spray pyrolysis	SWNTs	d = 0.8–1.2 nm	Co-Mo/silicon substrate, ethanol	[138]

### 5.1. Carbon source and inert gas

A variety of gaseous and liquid hydrocarbon feedstocks are used to produce nanotubes via thermal catalytic decomposition. The kind of hydrocarbon sources varies from light gases such as C_2_H_4_ and C_2_H_2_ to heavier aromatic liquids like xylene or benzene [67,86,87,88,89]. The carbon sources in form of liquids are vaporized before the entry into the reaction chamber. To avoid the oxidation of the carbon, the chamber is kept free of oxygen during the production process. Generally, continuous inert gas flow is supplied to the reaction chamber. Nitrogen, helium and argon are the most extensively used to create inert atmosphere [90].

Recently, also polymers were examined as precursors for the synthesis of graphitic carbon nanostructures. Carbon nanotubes, graphite films, carbon fibers were successfully prepared via pyrolysis of polyacrylonitrile (PAN) [91,92,93], and polypyrrole (PPy) [94,95,96,97]. These polymer materials opened a new way in the formation of carbonaceous structures, they demonstrated to be effective carbon sources.

### 5.2. Catalyst and support materials

Numerous catalysts [67,98,99,100] have been tried to improve the yield and the quality of CNT production. Also the effect of different supports [101,102,103,104,105,106] or buffer layers between the catalyst and Si wafers [107,108] has been investigated in detail. A wide range of transition metals individually or in mixture, and rare earth promoters have been tested for the synthesis of both SWNTs and MWNTs by CVD [109]. It is reported that the rare earth oxides can improve the activity and selectivity as potential co-catalysts in several catalytic materials. The selection of the metallic catalyst may affect the growth and morphology of the nanotubes. The most common metals found to be successful in the growth of carbon nanotubes are Fe, Co, and Ni. Fe/Mo bimetallic catalyst supported on MgO was found to be the most effective catalyst combination in the synthesis of single- and double-walled carbon nanotubes [110,111,112,113]. MgO support is useful in the production of nanotubes because it can be removed by a simple acidic treatment while other supports require a harsh hydrofluoric treatment.

**Figure 12:**
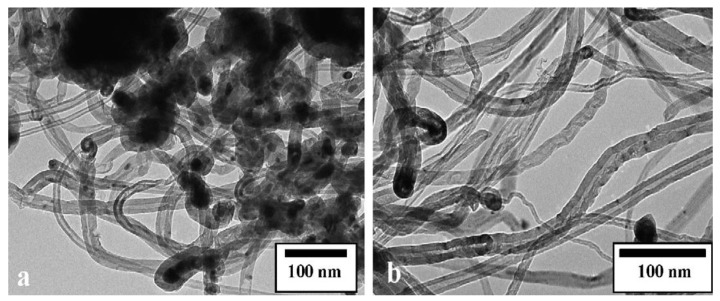
Carbon deposit produced on K-doped catalysts using acetylene as carbon source at 720 °C. (a) Co(-K)/CaCO_3_ catalyst. (b) Co-Fe(-K)/13X catalysts. Ref [114].

K-doped supported catalysts were examined in the CVD synthesis of multi-walled carbon nanotubes (Figure 12) [114]. Co and Co/Fe metal catalyst supported on zeolite and CaCO_3_ doped with different kinds of K-containing chemicals were applied. It was observed that bimetallic K-doped catalysts with higher Fe content demonstrate significantly higher activity than monometallic ones independently from the support material. The quality of the produced material was better in the case of zeolite when bundle-like structures of CNTs were formed. The best amount of potassium was established in 3 wt %. It was also observed that the kind of K-containing material influences the activity of the catalyst. The preparation method of the catalyst is an important step. Very low carbon deposits were obtained with carbon nanotubes of unusual shapes applying mechanical mixing of the catalyst components.

Natural minerals such as forsterite, diopside, quartz, magnesite (MgCO_3_) and brucite (Mg(OH)_2_) crystals were used as catalyst in the experiments of Japanese researchers [115] for the production of carbon nanotubes. Since these minerals contain certain quantity of Fe as impurity, it is utilized as metal catalyst. The syntheses were carried out with a mixture of CH_4_ and Ar gases at 800–900 °C. Single-walled carbon nanotubes (SWNTs) were successfully grown on magnesite crystal demonstrating the possibility of naturally occurring SWNTs. The Raman spectrum demonstrated strong G-bands at about 1590 and 1560 cm^-1^ and weak D-band at about 1330 cm^-1^ indicating the high quality of the obtained material. Different characterization results revealed that the SWNTs diameters are about 1–1.8 nm. The authors suppose that it is plausible that Fe atoms included in the CVD-treated magnesite sample diffuse to the surface of it, forming Fe clusters or nanoparticles that may act as a catalyst for SWCNT formation. However, the precise mechanism remains to be clarified.

A bundle of aligned carbon nanotubes were synthesized within the pores of an alumina membrane applying ethylene as carbon source without any metal catalyst [116]. In the absence of transition metals nanotubes are obtained as a print of alumina membrane showing a narrow diameter distribution. Wall thickness was controlled by varying the reaction time, while the tube length by the thickness of alumina membrane. 

Quite pure MWNTs in large quantities can be produced by the CVD method of methane on Co-Mo/MgO catalysts [117] or Ni/Mo, Y/Mo and Ni-Y/Mo systems [118].

### 5.3. The substrate

Recently used catalyst supports are in the form of different substrates. These catalysts permit controlling the metal nanoparticle sizes, therefore, the nanotube diameter and also the growth of aligned CNTs as a function of the particle dispersion. Hence the preparation of the substrate and the use of the catalyst deserves special attention, because they determine the structure of the tubes. The substrates used are silicon, glass and alumina. In the case of a Si substrate, the formation of metal-silicide, as a result of the preheating treatment, complicates the synthesis process. Buffer layers of Al, Ti or SiO_2_ have been previously used to prevent the formation of the metal-silicide. The catalysts are metal nanoparticles, like Fe, Co and Ni, which can be deposited on the substrates from solution, electron beam evaporation or by physical sputtering. The nanotube diameter depends on the catalyst particle size, therefore, the catalyst deposition technique. The ability to control the particle size is critical to develop nanodevices. Porous silicon is an ideal substrate for growing self-oriented nanotubes on large surfaces. It is proved that nanotubes grow on this substrate in higher amount and they are better aligned than on plain silicon. Hexagonal close packed nano-channel alumina templates with a Co-Ni catalyst is another substrate, where well-graphitized free-standing nanotube arrays can be grown. An important advantage of the template method is that the nanotubes prepared in this way can be diameter-controlled and well defined [119].

**Figure 13:**
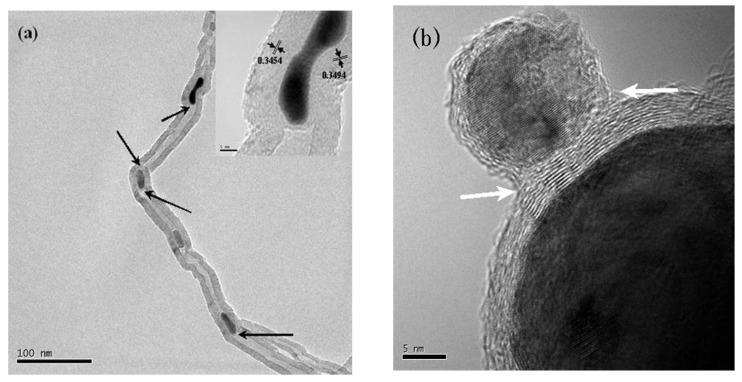
TEM images of products obtained on Ni/Y/Cu catalyst by decomposition of methane at 600 °C. (a) Metal filled carbon nanotube. (b) Onion-like carbon nanospheres. Ref [121].

**Figure 14:**
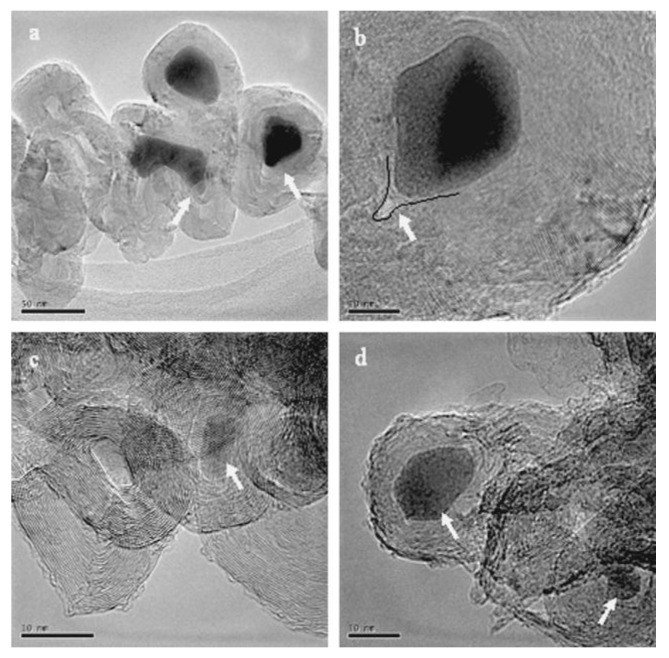
TEM micrographs of carbon onions produced on Ni/Y/Cu catalyst by decomposition of CH_4_ at different temperatures. (a,b) 600 °C (c,d) 700 °C Ref [123].

CNTs and carbon onions were prepared using Ni/Al catalyst with different proportions of each component [120,121]. Metal filled and bamboo-like carbon nanotubes (Figure 13), and carbon onions (Figure 14) were synthesized on the new copper-supported Ni/Y composite catalyst by decomposition of methane at 600 °C [122,123]. 

Commercial electrolytic copper powder was used as alternative support, which could open a new route for the fabrication of the CNTs *in situ* reinforced copper matrix composites. It is worth to note that the Ni/Cu catalyst is totally inactive resulting in almost no carbon deposition. Yttrium doped in nickel plays significant role in keeping the activity of the catalyst by resisting or postponing the interaction between Ni and Cu. Yttrium can assist CNTs and carbon onions synthesis by improving the activity and selectivity of the catalyst nanoparticles. Both ends of the CNTs are closed and traces of onion-like carbon nanospheres are found on them (CNTs). Furthermore, the presence of onion-like carbon nanospheres decreases as the reaction time increases, and meanwhile, carbon nanotubes become more abundant and longer. Thus, a sphere-tube mode for the CNT synthesis is proposed, which offers a new visual angle to understand the complicated mechanism of CNT growth. The diameter of bamboo-shaped CNTs was found to be in the range of 7–18 nm and those of carbon onions in the range of 10–90 nm. The morphologies of the carbon products will be changed along with the temperature increase: carbon nanotubes at 500 °C, carbon onions at 600 and 700 °C.

**Figure 15:**
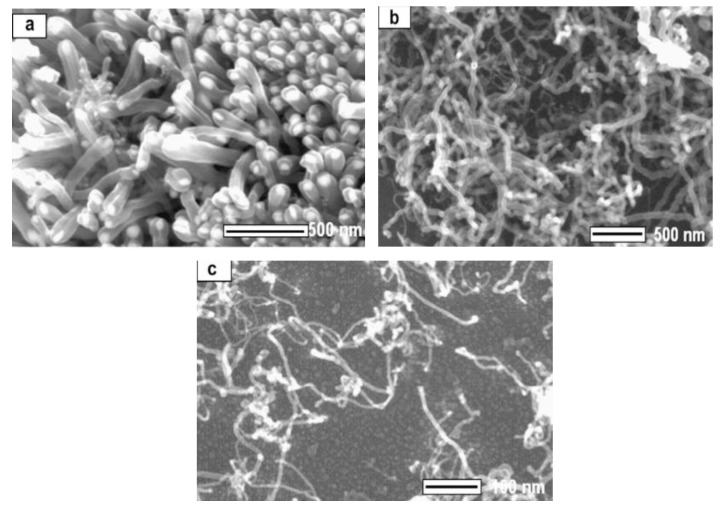
SEM photos of CNTs with various diameters produced on silicon substrate with different Co film thickness decomposing methane at 700 °C. (a) Film thickness 10 nm - tube diameter d = 80 nm. (b) Film thickness 5 nm – tube diameter d = 20 nm. (c) Film thickness 2 nm – tube diameter d = 5–8 nm. Ref [124].

Guo *et al*. [124] observed the fact that the diameter of the carbon nanotubes depend on the thickness of the deposited metal film on the substrate (Figure 15). With this parameter the particle size of the catalyst can be controlled, thus it is an important factor in the growth process escpecially for the production of SWNTs. For instance, thickness of 2 nm, 5 nm, and 10 nm samples resulted in formation of CNTs with diameter of 5–8 nm, 20 nm, and 80 nm, respectively. The nanotubes are aligned when they are large in diameter. When nanotubes are smaller than a certain size, they grew across the surface.

### 5.4. Gas phase metal catalyst

Generally, the metal catalysts are deposited or embedded on the substrate before the deposition of the carbon is started. Another method is to use a gas phase for introducing the catalyst, in which both the catalyst and the hydrocarbon gas are fed into a furnace followed by the catalytic reaction in the gas phase. The latter method is suitable for large-scale synthesis, because the nanotubes are free from catalytic supports and the reaction can be operated continuously [119].

The injection CVD or spray-pyrolysis method involves pumping or spraying a metallocene–hydrocarbon solution into a suitable furnace. In contrast to the usual CVD, the injection CVD method does not need catalyst synthesis step, since the catalytic particles are generated *in situ* continuously throughout the entire growth cycle. This gives the possibility to scale up the method for continuous or semicontinuous production. 

Horvath *et al*. [33] investigated the influence of metallocenes (ferrocene, cobaltocene and nickelocene) and various hydrocarbons (benzene, toluene, xylene, cyclohexane, cyclohexanone, n-hexane, n-heptane, n-octane and n-pentane) on the quality and quantity of the nanotubes grown by spray-pyrolysis. MWCNTs were produced with maximum yield when ferrocene–nickelocene catalyst mixture was used and xylene was found to be the most efficient carbon source. The samples contain mainly straight nanotubes and negligible amount of amorphous carbon and thick nanotubes with many defects.

**Figure 16:**
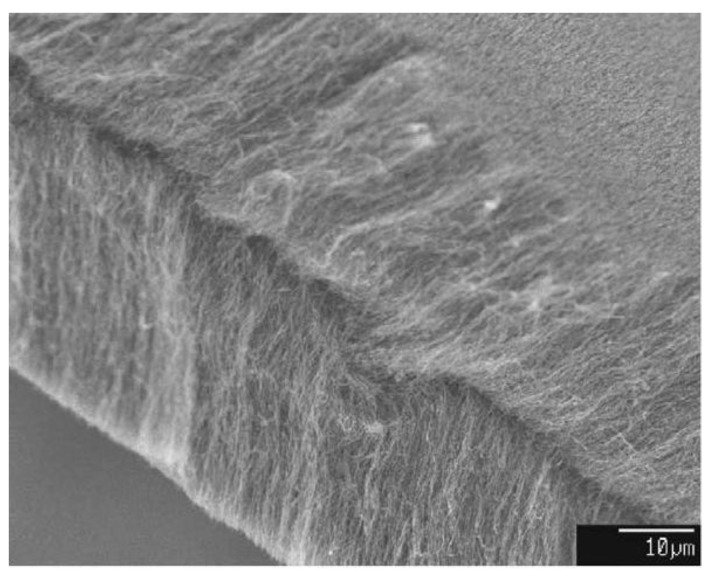
Aligned MWNT film grown at 740 °C on a quartz substrate by injection of a ferrocene/toluene solution. Ref [125].

Aligned MWNT films (Figure 16) were grown on the quartz substrate by an injection of a ferrocene/toluene solution into the reaction system [125]. The growth of well aligned MWNT films was observed at synthesis temperatures between 590 °C and 850 °C. Applying higher temperature unaligned nanotubes with diameter about 46 nm were produced. Increasing the injection time longer tubes were formed. Increasing the ferrocene concentration the tube diameter also increases and its distribution becomes broader, and higher formation of encapsulated nanoparticles is observed.

### 5.5. Different types of CVD

**Figure 17:**
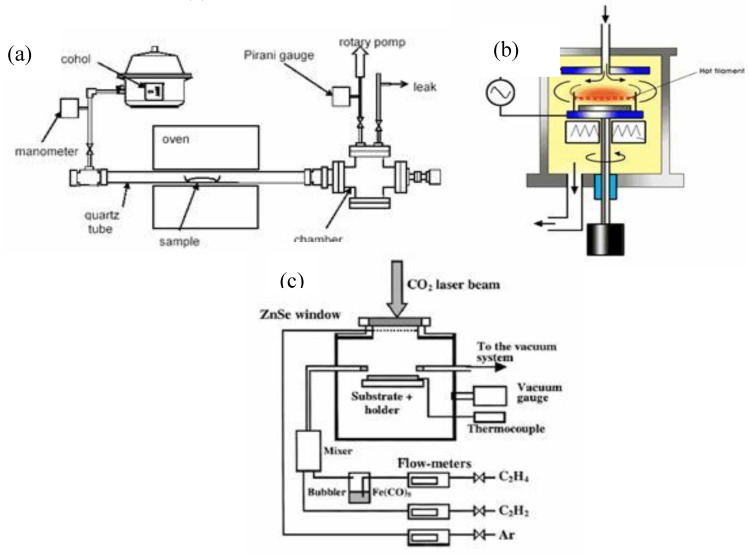
Schematic set-up of different types of CVD techniques. (a) Alcohol CVD. (b) Plasma-enhanced CVD. (c) Laser assistedCVD.

Recently, the commonly used thermal CVD tends to be replaced or increased the effect of hydrocarbon decomposition by hot-filament (HF-CVD), plasma-enhanced (PE-CVD), microwave-plasma (MP-CVD) or radiofrequency CVD technique [126,127]. Figure 17 shows some schematic apparatus of below mentioned CVD techiques. 

**Figure 18:**
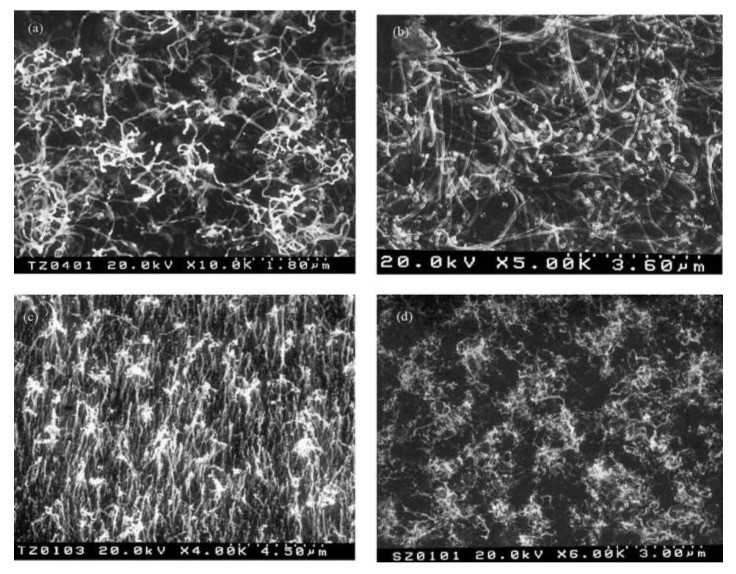
SEM images of carbon nanotubes produced by decomposition of CH_4_ on various catalysts. (a) Fe/ceramic catalyst at 650 °C. (b) Fe/Si catalyst at 600 °C. (c) Stainless steel at 600 °C. (d) Fe/Cr/glass at 650 °C. Ref [128].

Thermal CVD and PE-CVD are commonly used to grow aligned MWNTs and SWNTs on various substrates including Ni, Si, SiO_2_, Cu/Ti/Si, stainless steel, glass. Carbon nanotubes with different orientation can be produced by microwave plasma CVD using the same substrates as for PE-CVD. It was demonstrated that the nature of the substrate used influences the morphology of the carbon nanotubes formed. Using Fe/sapphire, Ni/Fe/glass and Cr/Fe/glass curved nanotubes were produced with inhomogeneous diameter and random orientation, while well aligned nanotubes were formed using stainless steel or Fe/Si as substrate (Figure 18). Some differences were observed also in this case, for example the amount of amorphous carbon was higher using stainless steel, while the samples obtained on Fe/Si substrate were pure, without amorphous carbon. Thus there is no need for the the purification step with this latter substrate [128].

Hot-filament enhanced CVD process is commonly used for depositing diamond films from a mixture of hydrocarbon/H_2_. The filament placed near the substrate acts as a local furnace, or a thermal source for decomposing the gas and often serves to heat the substrate as well. The use of carbon filament is more advantageous than the metallic one. While the latter can act as source of contamination, the carbon filament can act as catalyst to promote the amorphous carbon and CNT formation, it does not suffer from distortion due to the formation of carbide phases and also does not sag at operating temperatures (about 2000 °C). The influence of the Si support, gas ratio and the growth pressure was studied using hot-filament enhanced CVD method in the synthesis of carbon nanotubes [129]. 

The formation of only MWNTs was observed with average diameter more than 20 nm on catalyst film without Si support. Perpendicularly aligned MWNTs were grown on thin catalyst film while vertically aligned and highly dense MWNTs were achieved on thicker catalyst film. The tendency of growing vertically aligned MWCNTs is due to the van der Waals forces between the dense tubes. When using silica as support the formation of both SWNTs and MWNTs was observed depending on the reaction conditions. 

The formation of SWNTs were achieved at relatively low substrate temperature of 660 °C, low carbon supply, *i.e.* low C_2_H_2_ concentration and low reaction pressure. Using the optimal reaction conditions for SWNTs the tube diameter remains in the range of 0.65–1.55 nm. The gas ratio is an important synthesis parameter from point of view of the crystallinity degree of the tube which increases with decreasing the hydrocarbon concentration (optimum of C_2_H_2_ was found 2% or less in this case).

High yield growth of vertically aligned single-walled carbon nanotubes (SWNTs) and DWNT films was reported using a water-assisted [130], an oxygen-assisted plasma-enhanced [131], and microwave plasma-enhanced CVD technique (MWP-CVD) [132], in each case with the aid of different buffer layer. Vertically aligned SWNTs were fabricated by MWP-CVD using a mixture of Co-Ti nanoparticles deposited on the Si substrate without a buffer layer [133]. In the case of a Si substrate, metal-silicide could be formed as a result of the preheating treatment. Therefore, Ti nanoparticles were mixed with Co nanoparticles to prevent the formation of Co-silicides during the substrate heating process, and to maintain the size of the Co catalytic nanoparticles at approximately 1–2 nm. TEM observations reveal that most of CNTs are SWNTs and DWNTs free of metal particles. Their average diameter was established to be approximately 2 nm. When using a substrate with low-density catalytic nanoparticles, the CNT bundles had more space to grow and thereby grew in a curly fashion. With increasing cumulative density of catalytic nanoparticles, SWNT film with a high growth rate was attained due to the dense nucleation of CNTs from the doubled density of the catalytic nanoparticles. In contrast, with further increase in the cumulative catalytic nanoparticles density the growth of MWNTs was observed, probably due to the sintering of nanoparticles at the substrate temperature (about 700 °C), and thus the particles with large diameter (3–4 nm) were formed.

Alcohol-CVD is another type of commonly known CVD process. Here, the catalyst applied is often a solution of ferrocene and ethanol with different ratios. Various experiments were carried out studying the reaction parameters such as the type of catalyst, the effect of pressure, the furnace temperature [89,134,135,136]. Generally, the product obtained was high purity SWNTs using the optimal synthesis conditions. The effect of carrier gas flow rate was also studied, and it was observed that the length of CNTs increases by increasing the flow rate but only to a certain limit. Up to this maximum only the metal particle formation was observed. The ferrocene/ethanol ratio is another important parameter. When the concentration of ferrocene is too low a high amount of amorphous carbon is formed with few narrow tubes. In contrast, too high concentration causes the formation of high amount of nanoparticles, so the optimal concentration of ferrocene was established to be 1–1.5 wt %. No direct ferrocene concentration effect was found on the tube diameter [134]. The alcohol-CVD method was improved using high power laser pulse for the vaporization of solid metal target. Ethanol was chosen as carbon source and Co as metal catalyst [137]. SWNTs with diameter of 0.96–1.68 nm were synthesized. It was observed that SWNTs generated with different metal rods (Ni, Ni/Co, Fe) as catalysts have very similar diameter distribution.

The relationship among the nominal thickness of Co and Co-Mo catalysts, the structure of the catalyst particles, and the structure of carbon nanotubes growing by alcohol-CCVD method were investigated in the study of Maruyama *et al*. [138]. The authors suppose that different morphologies of CNTs such as individuals, random networks parallel to the substrate surface and vertically aligned forests of SWNTs and MWNTs can be produced by varying only the nominal thickness of metal catalyst in the same reaction conditions. These different morphologies at the same growth time were due to the different areal density rather than to the length of CNTs. With inceasing the nominal thickness of catalyst, the catalyst particles changed in diameter while their areal density remained relatively constant. The change in turn affected the areal density of CNTs and yielded the various morphologies. Longer growth time can cause also the changes from individual nanotubes to random network, and from random network to aligned forest. The optimal synthesis parameters were investigated for the production of vertically aligned SWNT forests [139]. The results were surprising because not only one but a few conditions were found which could be considered as optimal. For instance catalyst with Co/Mo = 1.6/1.0 ratio showed high catalytic activity at lower reaction temperature of 847 °C whereas it showed reduced activity at higher temperature of 947 °C. Oppositely, this reaction temperature enhanced the catalytic activity of the catalyst with Co/Mo ratio of 1/3. Similar tendency was observed for the flow rate. While the activity of the first mentioned catalyst was reduced, the activity of the latter one was enhanced at the same temperature of 847 °C. These observations are related to the presence of decomposition products, some of which become precursors for carbon nanotubes. Concerning the role of Mo, it is believed that it plays as co-catalyst to suppress the broadening of the SWNT diameter distribution. The SWNT diameter produced at the optimal synthesis conditions was about 2–3 nm.

Single-walled carbon nanotubes were synthesized by ultrasonic spray pyrolysis of ethanol utilizing Co-Mo/silicon catalyst [140]. This process has several advantages: (1) it is possible to grow carbon nanotubes without use of vacuum pump; (2) hydrogen is not necessary for the synthesis; (3) the set-up is simple; (4) the synthesis temperature is comparatively low. The formation of CNTs was highly affected by dipping time and by the catalyst concentration. Microscopic studies demonstrated the presence of catalyst agglomerations and also that larger diameter nanotubes were grown in longer dipping time. The diameter of the SWNTs was in the range of 0.8–1.2 nm.

### 5.6. Different kinds of carbonaceous materials

Single-walled carbon nanotubes were produced by CVD method at 900 °C using methane and Fe/Mo catalyst supported on the Si substrate. Individual nanotubes as well as tubes in bundles were formed with a diameter at about 1.15 nm [141]. Terrado *et al*. [142] reported the production of multiwall carbon nanotubes on quartz substrate using Co as metal catalyst. It was observed that the catalyst pretreatment with NH_3_ plays an important role in the growth process: without NH_3_ no CNTs were formed. The smallest Co particles and the highest particle density were achieved at the temperature at about 750–800 °C when thin MWNTs as well as aligned CNTs were grown. The employed growth temperature affects the diameter and the morphology of produced nanotubes. It was observed that MWNTs grown at 750 °C and 800 °C straighter and better aligned than those grown at higher temperatures. No significant MWNT production is observed in absence of ammonia or any other reductive pretreatment prior to the MWNT growth. However, when employing ammonia the efficient CNT production processes described above are achieved. The authors believe that the use of ammonia might improve the CNT production by keeping the Co nanoparticles active for nucleation. The atomic hydrogen that results from the ammonia decomposition might react with amorphous carbonaceous materials deposited on the catalyst surface, then forming volatile products that are easily removed from the catalyst and, therefore, keeping the metal surface clean.

Wei *et al*. [143] reported on the production of aligned CNTs synthesized by thermal CVD at atmospheric pressure using Co/SiO_2_ as catalyst and a mixture of gas Ar/H_2_ and NH_3_/N_2_. The influence of the Co film thickness and the flow rate of NH_3_ were tested. The density of the Co particle decreases by increasing the thickness of initially deposited Co film while their average diameter increases. The NH_3_ flow rate has significant effect on the Co particle size and density. The higher the flow rate, the higher the Co particle density and the smaller the Co particle diameter. Making comparison between the samples, it was observed that there is a critical NH_3_ flow rate or a threshold of NH_3_/C_2_H_2_ flow rate ratio (1.3–2) above which well aligned CNTs can be synthesized. The effect of NH_3_, as it was described in ref [140], is believed to keep the catalysts from being passivated by amorphous carbon because NH_3_ could act as a moderate reducing agent which reduces amorphous carbon at a much higher rate than it reduces CNTs. The density and morphology of produced CNTs can be controlled by varying the flow rates of NH_3_ and hydrocarbon.

**Figure 19:**
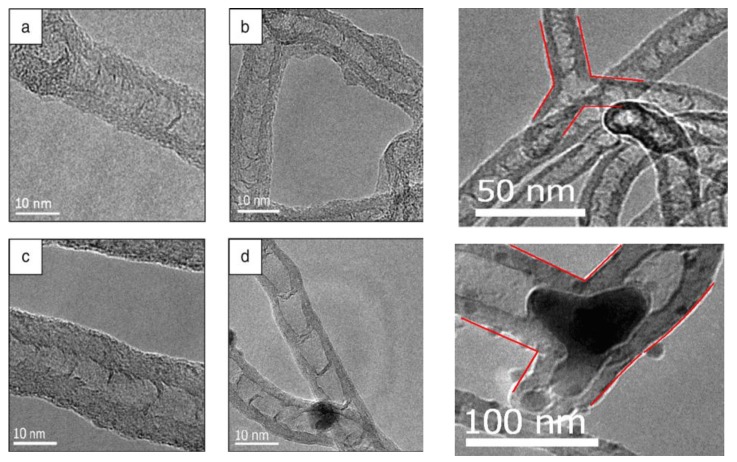
TEM micrographs of the nanotubes obtained on Cu/MgO catalyst using ethanol as carbon source at different synthesis temperatures: (a) 800, (b) 850, (c) 900, (d) 950 °C. Y-junctioned carbon nanotubes with (left) and without (right) metal particles. Ref [144].

Some studies (e.g., electron spin resonance) and applications (e.g., hyperthermia) require non-magnetic catalyst based samples. Thus, there is a growing interest in the use of catalysts which are non-magnetic and which can be removed relatively easily via less-aggressive purification procedures than currently employed (usually aggressive acid treatment). A potential candidate is copper, which has recently been demonstrated to work in laser ablation and chemical vapor deposition (CVD). It has been shown that the inclusion of Cu in the catalyst mix can play a very interesting role by promoting multi-branched types of carbon nanotubes. Borowiak and Rümmeli [144] reported on the application of copper as a catalyst on an oxide support (Cu-MgO) for the synthesis of CNTs in alcohol-CVD system. The use of copper as a highly efficient catalyst for CNT growth requires activation prior to the synthesis reaction. The key parameter of this activation process is a heat treatment of catalyst/support mixture in air. The present operation results in high yield of clean magnetically free bamboo-like multiwalled carbon nanotubes (BMWNT). Their mean diameter depends sensitively on the reaction temperature which allows the synthesis of nanotubes with tailored diameters. The mean outer diameter of the tubes increases with the increase of reaction temperature linearly from about 8 nm for the CNTs grown at 800 °C until just over 40 nm for the material grown at 950 °C. Raman studies demonstrated that the graphitic structures forming the BMWNT are less crystalline when formed at higher temperatures. The presence of Y-junctioned BMWNT was also observed (Figure 19). The use of Cu in conjunction with other catalysts has been shown to assist in the formation of carbon nanotubes with multibranched morphology [145].

Carbon spheres (CSs) can be fabricated by the methods that are normally used to synthesize carbon nanotubes. TEM examinations demonstrate that these carbon spheres are composed of crumpled graphene sheets. A simple chemical vapor deposition method was used to prepare carbon spheres from toluene without any catalysts [146]. The diameters of CSs are in the range of 60 nm to 1 µm and can be controlled by changing the composition and flow rate of a mixture of carrier gas.

The SEM examinations show that these carbon spheres have a ball-like and chain-like morphology, all of which have smooth surfaces and a quite uniform diameter. The flow rate of carrier gas plays an important role. It has been found that toluene would not be pyrolyzed completely if the flow rate of carrier gas is less than 20 mL/min or higher than 100 mL/min

**Figure 20:**
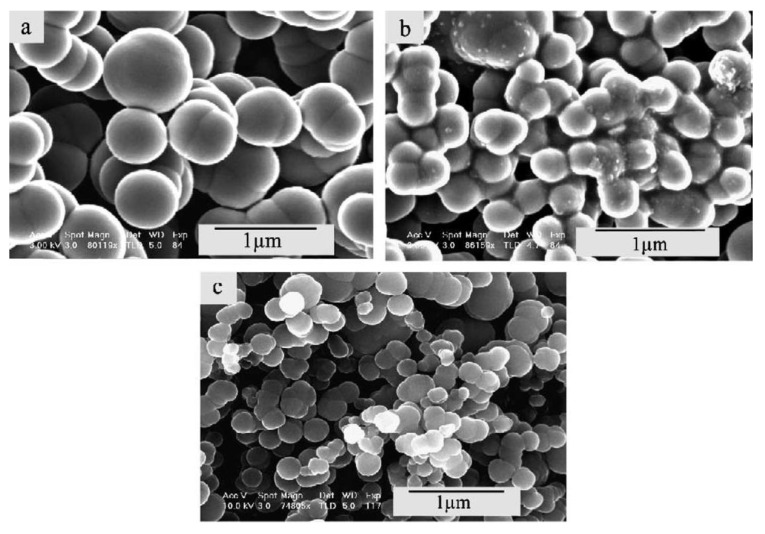
SEM images of carbon spheres obtained by pyrolysis of toluene without any catalyst using 80 mL/min flow rate of carrier gas (a) Ar; (b) H_2_; (c) N_2_. Ref [146].

The size distributions of carbon spheres obtained in N_2_, H_2_ and Ar atmospheres with the gas flow rate being kept at the same level of 80 mL/min are between 100–400, 200–600 and 400–900 nm, respectively (Figure 20). The effect of the carrier gas on the size and morphology of carbon spheres was further investigated in a mixed carrier gas, which was conducted by changing the ratio of argon to hydrogen. As the content of hydrogen in the gas mixture increases, the size distribution of carbon spheres becomes narrower, at the same time the diameters become smaller. In other words, the presence of argon in the mixed carrier gas makes the size or diameter distribution of carbon spheres broader. When an anodic aluminium oxide (AAO) template was used to fabricate carbon spheres, chain-like nanosized carbon spheres were obtained with diameters similar to the pore size of AAO template, e.g., 60 nm [146].

**Figure 21:**
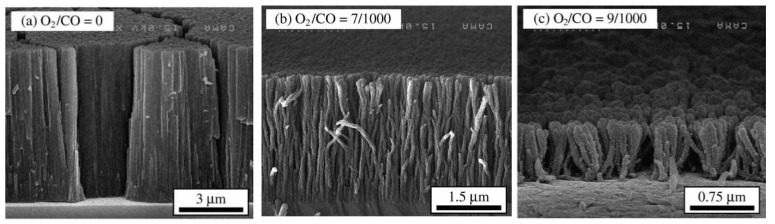
SEM images of carbon deposits on the glass substrates with different additional O_2_ flow rates. (a) O_2_/CO = 0. (b) O_2_/CO = 7/1000. (c) O_2_/CO = 9/1000. Ref [147].

Catalyst-free low-temperature growth of carbon nanofibers (CNFs) was performed by a microwave plasma-enhanced chemical vapour deposition using CO/Ar/O_2_ system. At the optimum oxygen concentration of O_2_/CO = 7/1000, vertically aligned CNFs with diameters between 50–100 nm can be synthesized at temperatures as low as 180 °C without any catalyst materials (Figure 21). It is believed that the addition of a small amount of O_2_ is the key for the synthesis of CNFs without catalyst, because it suppresses the isotropic deposition of amorphous carbon and assists the anisotropic linear growth of crystallized carbon deposit [147].

Without the addition of oxygen, pillar-like carbon films were formed. When a small amount of O_2_ was added to the CO plasma, the morphology of carbon films changed to fibrous structure.

At higher O_2_ flow rates, however, the deposition rate decreased and no carbon deposit could be observed. While O_2_/CO window for CNFs formation is shifted to higher O_2_ concentration side, the influence of oxygen addition on the morphology of carbon deposits is almost the same as the previously shown DC-PECVD results [148]. CNFs synthesized by DC-PECVD are quite straight while MW-PECVD grown ones are waved. The morphology of CNFs grown on the Si and CaF_2_ materials is almost the same as that obtained on the glass substrates. However, CNFs grown on polycarbonate has different morphology. The diameter of CNFs is increased and fiber bundling arises, the length of fiber is diminished.

Three-dimensional (3D) double-helix carbon microcoils (CMCs) were synthesized by catalytic pyrolysis of methane using Ni catalyst in hot wire CVD processes [149], using preheating method. Methane was preheated at 1500 °C in an upper reaction tube by a hot wire, and chemical vapor deposition of carbon then occurred at 700–770 °C in a lower reaction tube. Irregularly coiled CMCs were effectively synthesized. The diameter of carbon fibers from which the carbon coils were formed, was about 1 μm, the coil diameter was about 5–7 μm. The growth tip is very similar to those grown using acetylene, but less smooth in fiber surface. The CMCs produced in this way almost have an amorphous structure and can be graphitized at 2500 °C for 2.5 h. Microcoils with herringbone structure are obtained. The amount of coiled tubes increases with increasing the reaction time. When the CMCs growth temperature was slightly higher, such as 770 °C, instead of CMCs, the products were noodle-like or wave-like fibers. In this case, the anisotropy of the catalyst was weak, resulting in curling in two-dimension and formed wave-like fibers. The CMCs were less well-formed than conventional CMCs, and had a quite large coil pitch. This kind of CMC is expected to have large elasticity. These elastic CMCs have potential applications in electromagnetic wave absorption, in high performance fillers of polymer composites, such as CMCs/PU tactile sensor elements.

**Figure 22:**
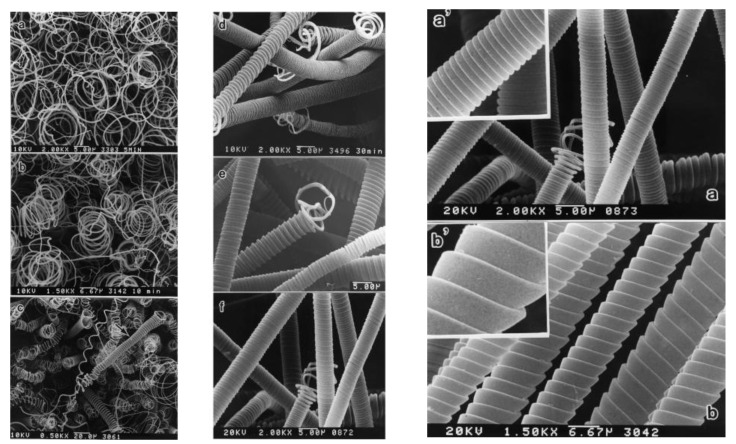
Growth stages of the carbon coils synthesized by the pyrolysis of C_2_H_2_ on Ni/quartz catalyst with small amount of thiophene impurity at 780 °C. Reaction time: (a) 5 min, (b) 10 min, (c) 15 min, (d) 30 min, (e) 60 min, (f) 120 min. Image in right side: Representative regular carbon coils. (a) Circular carbon coils. (b) Flat carbon coils. Ref [151].

Motojima *et al*. [150,151] reported about regularly microcoiled carbon fibers (Figure 22) synthesized in large scale with a high reproducibility by the catalytic pyrolysis of acetylene with a small amount of sulfur or phosphorus impurity utilizing various metal catalysts supported on quartz substrate. In this work, the effect of the catalyst preparation conditions, and the reaction conditions on the microcoil morphology and properties, as well as the growth mechanism were studied. However, when using hydrocarbons other than acetylene, the CMCs were rarely obtained under any reaction conditions. The authors proposed a 3D growth model based on the anisotropy for the carbon deposition among three crystal faces. 

Coiled carbon nanofibres were produced by CCVD decomposition of acetylene/thiophene mixture over submicron sized Ni powder as catalyst [152]. SEM analysis shows two kinds of coils morphology, one type with small tube diameter (~200 nm) and wavy morphology and the other group with relatively thicker diameter (~500–600 nm) and twisted morphology. 

**Figure 23:**
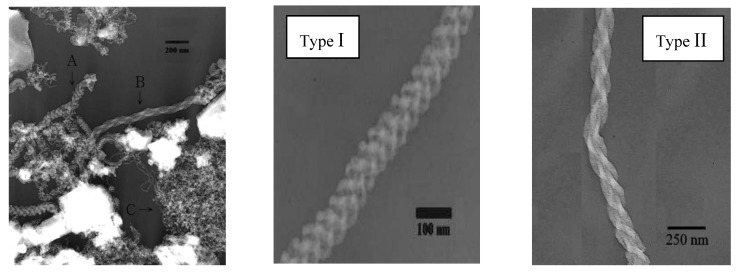
TEM figures showing three types of carbon nanofilaments grown on Pr/AlPO_4–_5 catalyst from decomposition of C_2_H_2_. Type I and II carbon nanoropes are marked as A and B, respectively. Carbon filaments in the area C mainly consist of irregularly curved single-stranded carbon nanotubes. Ref [153].

The authors believe that this difference can be caused by the catalytic anisotropy between the crystal faces of the catalyst grain which was proposed as a driving force behind the coiling factor. This anisotropy may be caused by the different chemical compositions of the crystal faces. The larger the anisotropy, the greater the degree of coiling/twisting and the coil diameter and this results in the twisted morphology of the coils. Triple-stranded helically coiled carbon nanoropes were prepared by high temperature decomposition of acetylene using prazeodymium catalyst supported on microporous aluminophosphate (AlPO_4_). Large numbers of irregularly curved nanofilaments with various shapes and forms were also found in the synthesis product but the triple-helices structures predominated (Figure 23). It should be noted that the formation of these triple-helices is quite sensitive to the reaction temperature, already they were observed to form in the temperature range of 695–705 °C utilizing the above described method [153].

**Figure 24:**
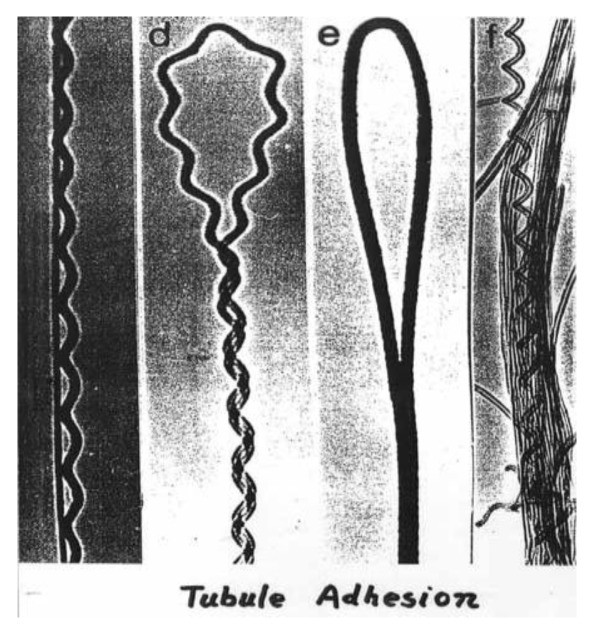
“The Belgian tubes” obtained from the decomposition of C_2_H_2_ on Co/SiO_2_ at 700 °C. Ref [4].

Helicoidal carbon nanotubes were first observed by Amelinckx *et al*. [4] in the CVD produced material and called “The Belgian Tubes” in 1994 (Figure 24).

**Figure 25:**
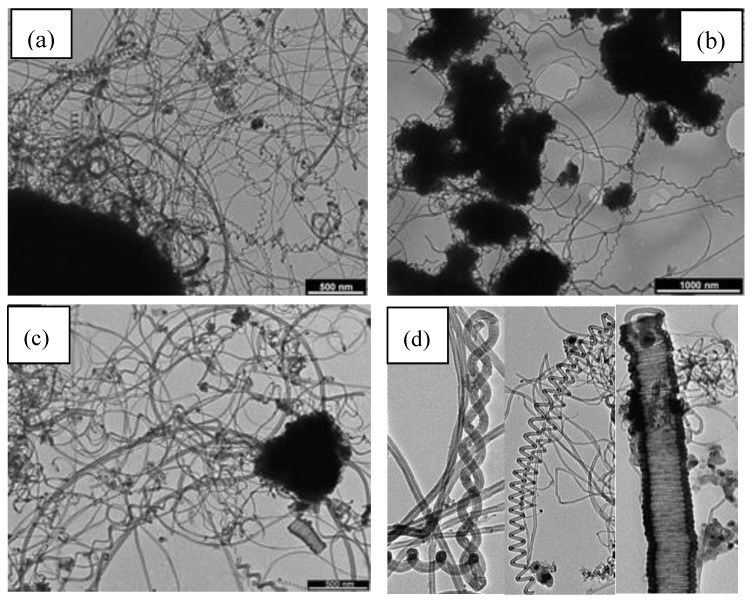
TEM images of coiled MWNTs synthesized C_2_H_2_ at 700 °C. (a) Co/SiO_2_ catalyst prepared by sol–gel technique. (b) CNTs on Co-Pr/SiO_2_ catalyst prepared by ion-adsorption precipitation. (c) CNTs on Co/SiO_2_ obtained by ion-adsorption. (d) Different types of coiled MWNTs. Ref [161].

The probability of the existence of coiled carbon structures was already predicted few years after the discovery of carbon nanotubes [154,155]. In the literature, there are many interesting theoretical studies about the formation and structural and electronic properties of these strange structures [156,157,158,159] but in reality their growth mechanism is not yet explored. Until today, lots of tentatives were made to reproduce these attracting carbon architectures with small success [159,160,161,162,163]. Up to now, coils were obtained in higher amount by decomposition of acetylene using Co/SiO_2_ and Co-Pr/SiO_2_ supported catalysts (Figure 25). 

It was observed that when the catalyst was treated at pH = 8–9, the amount of helicoidal nanotubes was higher in the product. It is supposed that at higher pH the shape of metal particles is more irregular and therefore during the reaction process crystal surfaces with different catalytic activities are created. The properties such as coil diameter and pitch depend on the preparation method and the type of catalysts. For example, carbon coils synthesized on Co catalyst have a pitch (the distance between adjacent corresponding points along the axis of the helix) in the range of 20–30 nm with coil diameter about 15–30 nm, some of which were larger about 100–150 nm coil diameter with a pitch of 10–15 nm. Coils prepared by Co/Pr catalyst have coil diameter about 30–150 nm and pitch about 50–100 nm. The helices observed have various morphology from regularly curved, spring-like to irregularly shaped ones. High-resolution TEM studies demonstrated two possible types for the coils structure, the one with continuous curvature and the other one when coiled nanotubes are composed of straight segments. The so called “stability islands” were established analyzing the coil pitch and coil diameter. These islands are the regions with higher number of coils with respect to other zones. The existence of these “stability islands” were confirmed by comparing the obtained results with the analysis of other research groups [161]. This fact can prove that the nature of coiled nanotubes is an inherent property and it is not influenced by external factors. However, the external factors contribute to the creation of the growth conditions. A Chinese research group [164] synthesized helically coiled carbon nanotubes in large scale through thermal reduction of ethyl ether. Double-helices and spring-like nanotubes were observed during microscopic examinations. Coiled carbon nanostructures with different morphologies such as slightly curved, spring-like, zig-zag shaped and loop-wire shaped, were prepared via reduced pressure CCVD on finely divided Co nanoparticles supported on silica [165]. In this work the influence of various parameters such as pH values, reaction pressure, flow rate of acetylene was also studied. The effect of pH on the formation of coils was observed again as in the work of Hernadi *et al*. [160], notably that the carbon yield decreased and the tube diameter and the ratio of coiled to straight tubes increased with increasing pH. The nanotubes yield and the fraction of coiled nanotubes increased with gas pressure and gas flow rate. Bai [166] reported the growth of nanofibre/nanotube coils by CVD on alumina substrate using acetylene as carbon source. The catalyst preparation way is the interesting step. The metal particles were deposited electrochemically on anodized porous alumina layer. Coiled nanotubes were produced with the following characteristics: coil diameter from 50 to 200 nm, pitch in the range of 130–160 nm, and tube diameter of 10–160 nm. Coiled nanofibres were obtained with following characteristics: coil diameter of 1–6 µm, pitch of 250 nm–1 µm and fibre diameter of 200–500 nm as a function of the catalyst particle size.

Cheng *et al*. [167] have successfully synthesized partially filled multi-walled carbon nanotubes using titanate-modified palygorskite as catalyst and acetylene as carbon source by chemical vapor deposition at high temperature. Different analysis results showed that the elongated nanomaterials encapsulated in the inner channels of the nanotubes were single crystalline Fe_3_C in form of nanowire. Not only the yield of carbon nanotubes but also the relative quantity of Fe_3_C nanowires was influenced by the reaction temperature. It was noted that the proportion of the filled CNTs at 800 °C was the highest. MWCNTs were also synthesized at 900 °C. Most of the CNTs were curved or bamboo-like. The inner cavities of the CNTs were rougher and the outer diameters were distinctly larger than those of CNTs fabricated at lower temperatures. In contrast to those prepared at 800 °C, there were fewer filled nanotubes and the nanowires were shorter. It has been suggested that materials encapsulated into the hollow cavities of CNTs would cause significant change in the physical properties of CNTs. Metal-filled CNTs being possibly used as heterogeneous catalysts and ferromagnetic nanoparticles (e.g., Fe, Co or Ni) are attractive for developing recording media due to their unique magnetic properties. Moreover, CNTs filled with metals or metallic compounds can produce nanoscale conductors, semiconductors and composites.

## 6. Hydrothermal Synthesis

Sonochemical/hydrothermal technique is another synthesis method wich is successful for the preparation of different carbonaceous nanoarchitectures such as nano-onions, nanorods, nanowires, nanobelts, MWNTs. This process has many advantages in comparison with other methods: i) the starting materials are easy to obtain and are stable in ambient temperature; ii) it is low temperature process (about 150–180 °C); iii) there is no hydrocarbon or carrier gas necessary for the operation. MWNTs were produced by hydrothermal processing where a mixture of polyethylene and water with a Ni catalyst is heated from 700 to 800 °C under 60–100 MPa pressure [168]. Both closed and open end multiwall carbon nanotubes with the wall thickness from several to more than 100 carbon layers were produced. An important feature of hydrothermal nanotubes is the small wall thickness and large inner core diameter, 20–800 nm. Graphitic carbon nanotubes were synthesized by the same research group using ethylene glycol (C_2_H_4_O_2_) solution in the presence of Ni catalyst at 730–800 °C under 60–100 MPa pressure [169]. TEM analysis shows that these carbon nanotubes have long and wide internal channels and Ni inclusions in the tips. Typically, hydrothermal nanotubes have wall thickness 7–25 nm and outer diameter of 50–150 nm. Thin-wall carbon tubes with internal diameters from 10–1000 nm have been also produced. During growth of a tube, the synthesis fluid, which is a supercritical mixture of CO, CO_2_, H_2_O, H_2_, and CH_4_ enters the tube.

Manafi *et al.* [170] have prepared large quantity of carbon nanotubes using sonochemical/hydrothermal method. 5 mol/l NaOH aqueous solution of dichloromethane, C°Cl_2_ and metallic Li was used as starting materials. The hydrothermal synthesis was conducted at 150–160 °C for 24 h. The nanotubes produced in this way were about 60 nm in diameter and 2–5 µm long. Uniformly distributed catalyst nanoparticles were observed by SEM analysis as a result of the ultrasonic pre-treatment of the starting solution.

Multiwall carbon nanocells and multiwall carbon nanotubes have been artificially grown in hydrothermal fluids from amorphous carbon, at temperatures below 800 °C, in the absence of metal catalysts [171]. Carbon nanocells were formed by interconnecting multiwalls of graphitic carbon at 600 °C. The bulk made of connected hollow spherical cells appears macroscopically as disordered carbon. The nanocells have diameters smaller than 100 nm, with outer diameters ranging from 15 to 100 nm, and internal cavities with diameters from 10 to 80 nm. The nanotubes observed in the sample have diameters in the range of tens and length in the range of hundreds of nanometers.

The spontaneous formation of short nanotubes and nano-onions occurs from nanoporous carbon in the presence of elemental Cs at temperatures as low as 50 °C [172]. Microscopic examinations showed that the carbon nanoparticles were more ordered, as well as they were present in larger numbers in the materials heated to 350 and 500 °C following addition of cesium. Carbon nanopolyhedra, tubes and onions were observed even in the sample heated to only 50 °C.

## 7. Electrolysis

Electrolysis is a less common method for CNT production which was developed by Hsu *et al*. in 1995 [173]. The main point of this method is electrowinning of alkali (Li, K, Na) or alkaline-earth (Mg, Ca) metals from their chloride salts on a graphite cathode followed by the formation of carbon nanotubes by the interaction of the metal being deposited with the cathode. A different optimum temperature for nanotube production has been found for each electrolyte composition, with the purity decreasing significantly on either side of this optimum temperature. For NaCl and LiCl electrolytes, the optimum temperature was just above the melting point of the salt. After the electrolysis the carbonaceous material is extracted by dissolving the ionic salt in distilled water and separating the dispersion by filtration. The cathode erodes during the electrolysis and the electrolytic products are a mixture of CNTs and a large proportion of carbon nanoparticles of different structures originating from the graphite cathode, carbon encapsulated metal particles, amorphous carbon and carbon filaments. The nanotubes produced are usually multi-walled; however, Bai *et al.* have grown SWNTs [174]. They prepared nanotubes by electrolytic conversion of graphite to carbon nanotubes in fused NaCl at 810 °C using Ar as inert gas. SWNTs are estimated to be 1.3–1.6 nm in diameter. This is comparable to the SWNTs obtained by other methods. Adding less than 1 wt % of metals or other salts of low melting point to the electrolyte, such as SnCl_2_, Sn, Pb, Bi or PbCl_2_, results in the formation of metal nanowires and filled carbon nanotubes. MWNTs possess diameters of 10–20 nm, consist of only a few walls, e.g., 10–15, and are estimated to be >500 nm long. MWNTs occur predominantly in entangled bundles also containing amorphous carbon, spherical carbon particles and metal encapsulated particles.

This method is unique compared with other production methods because it occurs in the condensed phase and uses graphite as the feedstock at relatively low temperatures. The advantages of this method are: i) apparatus simplicity, ii) possibility to control the synthesis process by the electrolysis modes, iii) use of cheap raw materials, iv) low energy consumption for electrolysis, v) possibility to control the product structures and morphologies as well as carbon phases doping in one step by means of optimization of electrolysis conditions and electrolytic bath composition [16,175,176].

A novel electrolytic synthesis method for carbon nanomaterial generation from ionic melts is being developed by Novoselova *et al*. [176]. The basis of the proposed method is the process of cathodic reduction of CO_2_ to elemental carbon on metallic electrodes. So in this method, a new condensed carbon phase is generated on the cathode from a liquid molten salt phase which contains dissolved carbonic acid by electrochemical reactions. Ternary mixture of alkali metal chlorides (NaCl:KCl:CsCl) was used as base electrolyte. The main products were MWNTs generally curved in form and often agglomerated into bundles. The outer tube diameter was in the range of 5–250 nm and the inner tube diameter was in the range of 2–140 nm. The nanotubes were partially filled with electrolyte salt. It was observed that when the current density increases the CNT diameter decreases and at the same time the carbon yield and the proportion of CNTs in the total mass of this product increase.

Nanotubes were also produced electrolytically at temperature as low as -40 °C in liquid NH_3_ from C_2_H_2_ without a metal catalyst. Acetylene was prepared by hydrolysis of calcium carbide. Gaseous acetylene was passed through a concentrated iron chloride water solution to be purified then through a thick layer of fresh melted potassium hydroxide flakes to dry it out. Purified acetylene was dissolved in liquid ammonia. After electrolysis the immersed part of the cathode was covered by a light-gray porous layer with average thickness of 1–2 μm. Auger analysis of the film indicated pure area C with oxygen content less than 3 at %. The deposit mostly consisted of amorphous, graphitic and turbostratic C and multi-walled CNTs. The nanotubes were curled irregularly and gathered into bunches. They had an average diameter of 15 nm and exhibited a high aspect ratio (length/diameter) greater than 1000 [177].

## 8. Solar Technique

Another method which is still being explored is through solar energy. It was used only for fullerene production until 1996. The production of carbon nanotubes using concentrated solar light was described by Laplaze *et al.* [178]. The advantage of the solar method is the use of light as in laser ablation to induce the vaporization of the target and the possibility to control each synthesis parameter independently. The carbon and catalyst mixture was vaporized at the front face impinged by the incident concentrated solar energy. A high-flow vacuum pump maintained the pressure. The buffer gas (Ar or He) entered around the perimeter of the quartz window and swept its interior surface to prevent any condensation of the carbon and catalyst vapors. A parabolic mirror positioned above the chamber focused the collected sunlight on top of the target material. The vaporization temperature remained in the range 2627–2727 °C. The mixture (buffer gas+carbon and catalyst vapors) was then aspired between the target and the graphite tube to the back of the reactor. A water-cooled heat exchanger cooled the gas mixture before it entered a bag filter. The target rod was a graphite cylinder in which a compressed mixture of graphite and catalyst powders was inserted inside holes drilled through the rod surface [16]. Luxembourg and co-workers [179] have demonstrated the production of SWNTs in gram quantities by solar process using a 50 kW solar reactor. The experimental set-up was installed at the focus of the 1 MW solar furnace. The sample quality has been investigated with respect to process parameters including target length, buffer gas (He or Ar) and location of the samples collection. Both parameters strongly affect the quantity of SWNTs but the influence of the buffer gas is dominant. The quality of the produced material increased with the target length in helium while its effect in argon was not so relevant, and poor quality product was obtained. The best sample was synthesized using Ni-Co catalyst with 2 at % of each in He atmosphere with target length of 15 cm. The SWNTs diameter is in the range of 1.2–1.6 nm.

## 9. Conclusions

Among the various methods shown in this review the CVD method clearly emerges as the best one for large scale production of MWNTs. However, the production of SWNTs is still in the gram scale and the helical carbon nanotubes are only obtained together with linear CNTs.

Several companies offer their products that are essentially MWNTs. Nanocyl (Belgium) produces already quite pure nanotubes in the ton scale, Hyperion (USA) was one of the pioneering company and Nanopart (Belgium) produces helical CNTs. Many small companies are producing SWNTs the quality of which is rather poor because it is difficult to purify SWNTs. Let us note that most of the companies are settled in USA.

As the demand for the use of CNTs will increase, these companies will develop and offer CNTs not only in large amount but also in a pure state.
